# Axl-148b chimeric aptamers inhibit breast cancer and melanoma progression

**DOI:** 10.7150/ijbs.39768

**Published:** 2020-02-10

**Authors:** Lorena Quirico, Francesca Orso, Carla L. Esposito, Sofia Bertone, Roberto Coppo, Laura Conti, Silvia Catuogno, Federica Cavallo, Vittorio de Franciscis, Daniela Taverna

**Affiliations:** 1 Molecular Biotechnology Center (MBC), University of Torino, Torino, Italy; 2 Dept. Molecular Biotechnology and Health Sciences, University of Torino, Torino, Italy; 3 Center for Complex Systems in Molecular Biology and Medicine, University of Torino, Torino, Italy; 4 Institute of Endocrinology and Experimental Oncology, CNR, Napoli, Italy

**Keywords:** aptamer, miR-148b, AXL, targeted therapy, metastasis

## Abstract

microRNAs (miRNAs) are small non-coding RNAs acting as negative regulators of gene expression and involved in tumor progression. We recently showed that inhibition of the pro-metastatic miR-214 and simultaneous overexpression of its downstream player, the anti-metastatic miR-148b, strongly reduced metastasis formation. To explore the therapeutic potential of miR-148b, we generated a conjugated molecule aimed to target miR-148b expression selectively to tumor cells. Precisely, we linked miR-148b to GL21.T, an aptamer able to specifically bind to AXL, an oncogenic tyrosine kinase receptor highly expressed on cancer cells. Axl-148b conjugate was able to inhibit migration and invasion of AXL-positive, but not AXL-negative, cancer cells, demonstrating high efficacy and selectivity *in vitro*. In parallel, expression of ALCAM and ITGA5, two miR-148b direct targets, was reduced. More importantly, axl-148b chimeric aptamers were able to inhibit formation and growth of 3D-mammospheres, to induce necrosis and apoptosis of treated xenotransplants, as well as to block breast cancer and melanoma dissemination and metastatization in mice. Relevantly, axl aptamer acted as specific delivery tool for miR-148b, but it also actively contributed to inhibit metastasis formation, together with miR-148b. In conclusion, our data show that axl-148b conjugate is able to inhibit tumor progression in an axl- and miR-148b-dependent manner, suggesting its potential development as therapeutic molecule.

## Background

Cancer is a multistep disease in which normal cells undergo genetic changes and start to proliferate abnormally within a microenvironment made of stroma cells embedded in a remodeled extracellular matrix invaded by immune cells. When some tumor cells acquire the capability to invade adjacent tissues, intravasate, roll through the vasculature, arrest in capillaries, extravasate into the surrounding tissue parenchyma, distant metastases form 1. Considering that metastasis formation is responsible for more than 90% of cancer-associated deaths, it is mandatory to develop new therapies with suitable specific delivery to metastatic cells.

miRNAs are small non-coding RNAs that have emerged as negative post-transcriptional regulators of their target genes. Aberrant expression of miRNAs in tumor formation and progression is well-known and new data are coming out continuously. The first report about the role of miRNAs in cancer appeared in 2002 focusing on the consequences of miR-15 and miR-16 deletion in Chronic Lymphocytic Leukemia (CLL) 2; since then, more than 32,500 studies have been published. Among many others, let-7, miR-29 family, miR-34 and miR-148b have been found to act as “tumor or metastasis” suppressors while the miRNA clusters 17-92, miR-21, miR-10b and miR-214 were shown to promote tumor growth or dissemination depending on the tumor context 3-8. Because of miRNA deregulation in tumor progression, cancer therapies based on miRNA targeting have been developed and used in clinical trials 9, 10, even if a main hurdle for their clinical translation still remains the development of selective and effective delivery strategies. We previously demonstrated that miR-148b controls breast cancer progression and metastasis by coordinating a large number of targets, among them the integrin ITGA5, its downstream players ROCK1 and PIK3CA/p110α 7, and the cell-to-cell adhesion molecule ALCAM 5. In addition, we proved that miR-148b is downregulated by the pro-metastatic miR-214, which therefore works in an antagonistic manner in the control of breast cancer and melanoma dissemination. Importantly, we evidenced that the simultaneous inhibition of miR-214 and the overexpression of miR-148b in tumor cells led to metastasis inhibition in an accentuated manner, compared to single modulations 8, giving hope for the application of multiple miRNA-based therapeutic interventions. On this line, we recently demonstrated that modified anti-miR-214 oligonucleotides, injected in the blood circulation of mice bearing miR-214 overexpressing tumors or metastases, successfully inhibited tumor cell dissemination 11.

With the intent to develop miRNA-based therapeutic tools avoiding the occurrence of undesired side effects in normal tissues, here we generated an aptamer-based chimeric molecule able to specifically deliver miR-148b to tumor cells. Aptamers are single-stranded oligonucleotides that bind to specific targets, usually transmembrane molecules, with high affinity and selectivity leading to signaling inhibition 12. However, apart from acting on their targets, aptamers can be used as carriers to convey therapeutic tools (chemotherapy drugs, siRNAs and miRNAs) to cells in a specific manner 13-17. Since most tumor cells overexpress AXL, a tyrosine kinase receptor with oncogenic functions, an RNA aptamer against AXL, GL21.T, has been developed and used to block AXL downstream signaling 18 and to selectively deliver small non-coding RNAs 19-21. In our work, we conjugated miR-148b to GL21.T to generate an axl-miR-148b conjugate or chimeric aptamer (from now on, briefly called axl-148b), and obtained a specific increase of miR-148b in treated AXL-expressing cells with the consequent silencing of ALCAM and ITGA5, two miR-148b direct targets. No effect was observed in AXL negative cells. More importantly, axl-148b chimeric aptamer was able to strongly inhibit metastatic traits *in vitro,* to induce apoptosis and necrosis in primary tumors and to block tumor cell dissemination and metastasis formation in mice, when injected intratumorally, compared to unrelated sequences, therefore giving hope for further therapeutic development.

## Methods

### Cell culture

MA-2 and MC-1 cells were kindly provided by L. Xu and R.O. Hynes 22 and maintained as in 4, 5. MDAMB231, SKBR3 and A549 were from American Type Culture Collection, instead, 4175-TGL were generously obtained from J. Massagué 23 and maintained in standard conditions. Human Umbilical Vein Endothelial Cells (HUVECs) were kindly provided by M.F. Brizzi and maintained as in 4, 5.

### Reagents and antibodies

miR precursors: Pre-miR™ miRNA Precursor Negative Control #1, Pre-miR™ miRNA Precursor hsa-miR-148b (PM10264) (Applied Biosystems). TaqMan® MicroRNA assays for miRNA detection: Hsa-miR-148b-3p ID 000471, U6 snRNA ID001973, U44 snRNA ID001904 (Applied Biosystems). Quantitect Primer Assay: 218300Axl ID 33000 (Qiagen). Qiagen miScript-SYBR Green PCR Kit and miScript Primer Assay: hsa-let-7g ID 1 (Qiagen). Primary antibodies: anti-Cleaved Caspase-3 (Asp175) #9661 (Cell Signaling Technology), anti-Ki67 ab15580 (Abcam), anti-ITGA5 pAb RM10 kindly provided by G. Tarone laboratory (Molecular Biotechnology Center, University of Torino), anti-CD166/ALCAM mAb MOG/07 (Novocastra Laboratories), anti-GAPDH pAb V-18, anti-ACTIN I-19 pAb (all form Santa Cruz Biotechnology), anti-AXL (R&D Systems). Secondary antibodies: HRP-conjugated goat anti-mouse IgG, goat anti-rabbit IgG (Santa Cruz Biotechnology).

### Transient transfections and stable cell lines

To obtain transient miRNA expression, cells were plated in 6-well plates at 80% confluency and transfected with 75 nmol/l of pre-miR 24 h later using LipofectamineTM2000 (Invitrogen Life Technologies) reagent, according to the manufacturer's instructions. Cells were tested for miRNA overexpression 48 h later. The human pre-miR-148b sequence (a 306-bp fragment containing the pre-miR sequence) was amplified from genomic DNA (MDAMB231) and cloned into pLenti4/V5-empty (Open Biosystems, Huntsville, AL, USA) vector to obtain pLenti-4/V5-148b (still containing tRFP) vector. Stable cell lines were generated by lentiviral infection. Lentiviruses were produced by calcium phosphate transfection of 293T cells with 20 µg of specific vector together with 15 µg packaging (pCMVdR8.74) and 6 µg envelope (pMD2.G-VSVG) plasmids, according to D. Trono's laboratory protocol (École Polytechnique Fédérale de Lausanne, Lausanne, Switzerland; http://tronolab. epfl.ch). Supernatant was harvested 48 h posttransfection, filtered with 0.45 mm filters, diluted and used to infect 3.5x10^5^ cells in 6-well plates, in presence of 8 µg/ml Polybrene (Sigma-Aldrich, St Louis, MO).

### Aptamer-miRNA conjugate preparations and binding

To generate axl-148b chimeric aptamers, a precursor of miR-148b was conjugated to an anti-AXL aptamer, GL21.T 18, *via* sticky-end annealing. Briefly, the complementary miR-148b guide strand was annealed to the passenger strand. Then the passenger strand of miR-148b and the GL21.T, elongated at their 3' ends with complementary 17-mer sequences, were annealed through their sticky sequences. The RNA sequences used are:

GL21.T-sticky (AXL aptamer): 5'AUGAUCAAUCGCCUCAAUUCGACAGGAGGCUCACXXXXGUACAUUCUAGAUAGCC 3';

GL21.T scramble (scr):

5'GGCGCUAGAACCUUCUAAGCGAAUACAUUACCGCXXXXGUACAUUCUAGAUAGCC3';

miR-148b guide strand (3P):

5' AGUCAGUGCAUCACAGAACUUUGUCU**UU** 3';

miR-148b passenger strand (5P): 5'AGGUGAAGUUCUGUUAUACACUCAGGCUGGCUAUCUAGAAUGUAC 3';

Guide scr:

5' UCUACUGUCACUCAGUAGU 3'

Passenger scr sticky:

5' ACUACUGAGUGACAGUAGAGGCUAUCUAGAAUGUAC 3'

For let-7g sequences, see 19.

All RNAs included modified 2′-F pyrimidines (2'F-Py) to improve stability and were synthesized at the Synthetic and Biopolymer Chemistry Core, Beckman Research Institute City of Hope, Duarte, CA. miR-148b guide strand held two overhanging bases (**UU**) at the 3' end to favor Dicer processing 24. Sticky sequences, consisting of 2′-F-Py and 2'-0-Methylpurine2′ (2'OMe-Pu), are underlined. X indicates C3 carbon linkers. To prepare axl-148b, scr-148b or axl-let-7g conjugates: 1) miR-148b or let-7g passenger and guide strands were annealed following incubation in annealing buffer at 95 °C for 15 min, at 55 °C for 10 min and then at 37 °C for 20 min; 2) sticky or scramble aptamers were refolded (5 min 85 °C, 3 min on ice, 10 min at 37 °C); 3) equal amounts of sticky/scr aptamers and annealed passenger-guides were then annealed by incubating them together at 37 °C for 30 min. The annealing efficiency was controlled with non-denaturing polyacrylamide gel electrophoresis 25. For treatments, cells were plated in 24-well dishes at 80% confluency and treated 24 h later with the folded axl, axl-148b, scr-148b or axl/scr aptamers.

### Binding assay

Cells were seeded in 3.5-cm plates (2.0×10^5^ cells/plate) and the day after treated with 200 nmol/L axl-148b chimera for 30 min at 37 °C. Unbound RNA was removed by washing the cells 3 times with ice-cold Dulbecco's Phosphate-Buffered Saline (DPBS). Bound RNA was recovered by TRIzol containing 0.5 pmol/ml of CL4 aptamer (CL4: 5′ GCCUUAGUAACGUGCUUUGAUGUCGAUUCGACAGGAGGC 3′) as a reference. The amount of recovered RNA was determined by performing a two-step qRT-PCR. In step one, recovered RNA was used for the reverse transcription (RT) using MMuLV and specific 3′ primers as follows: GL21(3′) 5′ GTGAGCCTCCTGTCGA 3′; CL4(3′) 5′ GCCTCCTGTCGAATCG 3′. The protocol used for RT was: denaturation, 5 min at 65 °C; annealing, 5 min at 22 °C, extension, 30 min at 42 °C; followed by a final extension of 30 min at 48 °C and enzyme inactivation (5 min at 95 °C). In step two, the products from the RT reaction were amplified by PCR with the iQ SYBR Green Supermix (Bio-Rad, Hercules, CA, USA) with the following protocol: denaturation, 2 min at 95 °C, followed by 40 cycles of denaturation, 30 s at 95 °C; annealing 30 s at 55 °C; extension 30 s at 60 °C. A melting curve was previously performed by heating the samples from 60 °C to 95 °C. Primers used here were: GL21(5′) 5′ AGATCATGATCAAT CGCC 3′; GL21(3′) 5′ GTGAGCCTCCTGTCGA 3′; CL4(5′) 5′ GCCTTAGTAACGTGCTTT 3′; CL4(3′) 5′ GCCTCCTGTCGAATCG 3′. Data were normalized to CL4 (reference) and to cell number, as determined by counting cells cultured in conjunction with each experiment.

### RNA isolation and qRT-PCR for miRNA or mRNA detection

Total RNA was isolated from cells using TRIzol® Reagent (Invitrogen Life Technologies). qRT-PCRs for miRNA detection were performed with the indicated TaqMan MicroRNA Assays (Applied Biosystems) on 10 ng total RNA, according to the manufacturer's instructions. For mRNA detection, 1 μg of DNAse-treated RNA (RQ1 RNase-Free DNase, Promega, Madison, WI) was retrotranscribed with High-Capacity cDNA Reverse Transcription Kit (Thermo Fisher Scientific, Waltham, MA) and qRT-PCRs were carried out using gene-specific primers for mRNA detection, using a 7900HT Fast Real Time PCR System (Thermo Fisher Scientific). Quantitative normalization was performed on the expression of the RNU44 or U6 small nucleolar RNAs or of RRN18S, for miR or mRNA detection, respectively. The relative expression levels between samples were calculated using the comparative delta Ct (threshold cycle number) method (2-ΔΔCt) with a control sample as the reference point.

### Protein preparation and immunoblotting

Total protein extracts were obtained using a boiling buffer containing 0.125 M Tris/HCl, pH 6.8 and 2.5% Sodium Dodecyl Sulphate (SDS). In all, 10-60 μg of proteins were separated by SDS polyacrylamide gel electrophoresis (PAGE) and electroblotted onto nitrocellulose membranes (BioRad, Hercules, CA). Membranes were blocked in 5% non-fat milk phosphate-buffered saline (PBS)-Tween buffer (4.3 mM Sodium Phosphate, Dibasic Na_2_HPO_4_, 137 mM Sodium Chloride NaCl, 2.7 mM Potassium Chloride KCl, 1.4 mM Potassium Phosphate, Monobasic KH_2_PO_4_, pH 7.4, with 0.1% Tween-20) for 1 h at 37 °C, then incubated with appropriate primary and secondary antibodies in PBS-Tween buffer, respectively, overnight at 4°C or for 1 h at room temperature and visualized by enhanced chemiluminescence (ECL®, GE Healthcare).

### Proliferation and viability

5x10^3^ MA-2 or 4175-TGL cells/well were plated in 96-well plates in complete medium and starved for 24 h. Complete medium was then added and cells were allowed to grow for 1, 2, 3 and 4 and days, fixed with 2.5% glutaraldehyde and stained with 0.1% crystal violet. The dye was solubilized using 10% acetic acid and optical density was measured using Promega GloMax®-Multi Detection System (Promega, Madison, WI) at 600 nm wavelength. Viability assay was performed following manufacturer's instructions using the CellTiter-Glo® Cell Viability Assay and GloMax® Discover System (Promega).

### Migration, invasion and transendothelial migration transwell assays

To measure migration and matrigel invasion 7.5x10^4^ MA-2, 5x10^4^ 4175-TGL or A549, and 1x10^5^ SKBR3 were seeded in serum-free medium in the upper chambers of cell culture transwells with 8.0 μm pore size membrane (BD Biosciences, NJ), pre-coated or not with 4 μg/well growth factor reduced matrigel (BD Biosciences, NJ). The lower chambers were filled with complete growth medium. After 18h or 24h, the migrated cells on the lower side of the membrane were fixed in 2.5% glutaraldehyde, stained with 0.1% crystal violet and photographed using an Olympus IX70 microscope. For transendothelial migration assay 10^5^ HUVECs were seeded in complete medium in the upper part of transwell inserts with 5.0 μm pore size membrane (Costar, Corning Incorporated, NY) coated by 0.1% gelatin, and grown till confluency. Then, 5x10^4^ cells labeled with CellTracker™ Orange CMRA or Green CMFDA (Molecular Probes, Invitrogen Life Technologies, Carlsbad, CA), were seeded onto the HUVECs monolayer. 20h later HUVECs and non-transmigrated cells were removed and the red or green fluorescent cells that migrated on the lower side of the membrane were fixed in 4% paraformaldheyde and photographed using Zeiss Axiovert200M microscope. Migration, invasion and transendothelial migration were evaluated by measuring the area occupied by migrated cells using the ImageJ software (http://rsbweb.nih.gov/ij/).

### Mammosphere formation assays

Mammosphere formation assays were performed as in *https://www.stemcell.com/tumorsphere-culture-human-breast-cancer-cell-lines-lp.html* on poly-HEMA (poly-2-hydroxyethyl-methacrylate)-coated 24 well plates following two different protocols. In one case, single breast cancer cells (8x10^3^ cells/well for 4175-TGL; 1x10^4^ cells/well for SKBR3) were plated (day 0), maintained in suspension in MammoCult Medium (StemCell Technologies) and left untreated (controls = ctrl) or treated with 400 nmol/L of axl, axl-148b or scr-148b aptamers. Treatments were renewed at day 3 and 5 (200 nmol/L). At day 5 sphere size and number were evaluated using a Zeiss AxioObserver microscope (Zeiss) and ImageJ Software *(http://rsbweb.nih.gov/ij/).* For size evaluation, the long side of spheres was measured (length). For number evaluation, the total number of spheres present in 50 µl of volume was counted for each treatment. Alternatively, single cells were plated and maintained as above and mammospheres were dissociated at day 5, counted and plated again at the same density and similarly treated. Spheres were analyzed at day 12. In some cases, cells were labelled with PKH26 (Sigma, 10^-7^M, 5 min) at day 5 and the percentage (%) of PKH26 positive cells analyzed at day 12 by FACS following mammosphere dissociation to evaluate mammosphere formation and growth. A FACSCalibur cell analyser was used to evaluate PKH26^positive^ cells on the total pool (100%).

### Histology and immunohistochemistry

5 µm-thick tissue sections were generated from Formalin-Fixed Paraffin Embedded (FFPE) tumor samples and stained with Hematoxylin and Eosin (H&E) for standard histology observations. ImmunoHistoChemistry (IHC) stainings were also performed using an anti-Ki67, anti-Cleaved Caspase-3 or anti-AXL antibodies with the avidin-biotin-peroxidase techniques (Anti-Mouse HRP-DAB Cell & Tissue Staining Kit, R&D Systems). Slides were counterstained with Hematoxylin.

### Axl-148b chimeric aptamer stability in human serum

Conjugate stability was investigated by incubating the molecule in human serum (Type AB Human Serum, Euroclone Cat.ECS0219D) as in 25. Briefly, Axl-148b was incubated in 80% human serum for 1 h to 7 days. At each time point 8μl of 80% serum solution (equal to 32 pmol RNA) was withdrawn and incubated for 2 h at 37 °C with 1μl of proteinase K solution (600 mAU/ml). Then, 9μl of 1×TBE and 3μl of gel loading buffer (Invitrogen, Waltham, MA, USA) were added to samples and stored at -80 °C. All time-point samples were separated by electrophoresis into 10% non-denaturing polyacrylamide gel stained with ethidium bromide.

### *In vivo* tumor growth and metastasis assays

All experiments performed with live animals complied with ethical care. NOD/SCID/IL2R_null (NSG) mice were injected with 5× 10^6^ 4175-TGL or MA-2 tumor cells into the mammary fat-pad or subcutaneously into the flanks 5, 8. When tumors were palpable, animals were treated with PBS or axl or axl-148b or scr-148b solutions (300 pmol/injection, three injections a week). Mice were sacrified and dissected 11, 18 or 32 days after cell injections. Primary tumor weight/morphology and lung or liver metastases were evaluated 5, 8. Organ (liver, spleen, kidneys) size (weight) and morphology (H&E) were analyzed at the end point.

### Circulating tumor cell isolation

Circulating Tumor Cells (CTCs) were isolated as in 11. Briefly, blood was collected from heart-punctured mice and put in culture plates with normal medium for 3 days; subsequently, attached cells were washed and cultured in fresh medium in presence of puromycin to select puromycin-resistant tumor cells. 7 days later, cells were washed thoroughly to remove non-adherent cells, fixed with 2.5% glutaraldehyde and endogenous cell fluorescence was used for visualization at the fluorescent microscope.

### Statistical analysis

All results are shown as mean ± Standard Deviation (SD) or ± Standard Error of Mean (SEM), as indicated, and two-tailed Student's t test was used for comparisons. * = p<0.05; ** = p<0.01; *** = p<0.001 were considered to be statistically significant. ns = indicates a non-statistically significant p-value.

## Results

### Axl-148b conjugate inhibits tumor cell movement*,* but do not affect proliferation

We previously demonstrated the anti-metastatic properties of miR-148b in cancer cells 7, 8. With the purpose to evaluate the potential of miR-148b as therapeutic target and to obtain a specific delivery of this non-coding RNA to cancer cells, we generated a chimeric axl-miR-148b (axl-148b) molecule (Figure S1A). Precisely, we linked miR-148b to GL21.T 18, a previously well characterized aptamer able to bind AXL, a tyrosine kinase receptor with oncogenic functions, overexpressed on many tumor cells. The annealing efficiency and serum stability of axl-148b conjugate were verified by non-denaturing gel electrophoresis analysis (Figure S1B and C), and the binding to AXL was measured on AXL^+^ A549 cells, compared to GL21.T aptamer (Figure S1D). Effective annealing, proper serum stability and binding to the target cells were evidenced. Next, we evaluated miR-148b efficient delivery to AXL positive cells. As revealed by qRT-PCR analysis, A549 lung adenocarcinoma, MA-2 and MC-1 melanoma, MDAMB231 and 4175-TGL breast cancer cells express AXL (AXL^+^), while SKBR3 breast tumor cells do not (AXL^-^) (Figure S2A). Therefore, AXL^+^ or AXL^-^ cells were treated with chimeric axl-148b molecules and miR-148b levels evaluated by qRT-PCR analysis after 48 hours and compared to cells left untreated (control = ctrl) or treated with GL21.T aptamer alone or with an unrelated/scramble-miR-148b (scr-148b) conjugate. Alternatively, the same cells were transfected with pre-miR-148b (pre-148b) or pre-control (pre-ctrl). Relevantly, as shown in (Figure S2B-F and Table S1), an important overexpression of miR-148b-3p (indicated as miR-148b) was found in AXL^+^, but not in AXL^-^ cells compared to ctrl, GL21.T or scr-148b treated cells upon treatment with axl-148b chimeras and the increased expression was dose- and time-dependent (data not shown). Similarly, let-7g levels increased in A549 cells when another small RNA, let-7g, was linked to GL21.T (Figure S2G). A strong increase in miR-148b levels was instead observed for all cells transfected with pre-148b, but not with pre-ctrl, including AXL^-^ SKBR3 cells. Taken together, these results give hope for a proficient specific delivery of miRNAs in AXL^+^ cells using axl-miRNA conjugates.

**Figure 1:**
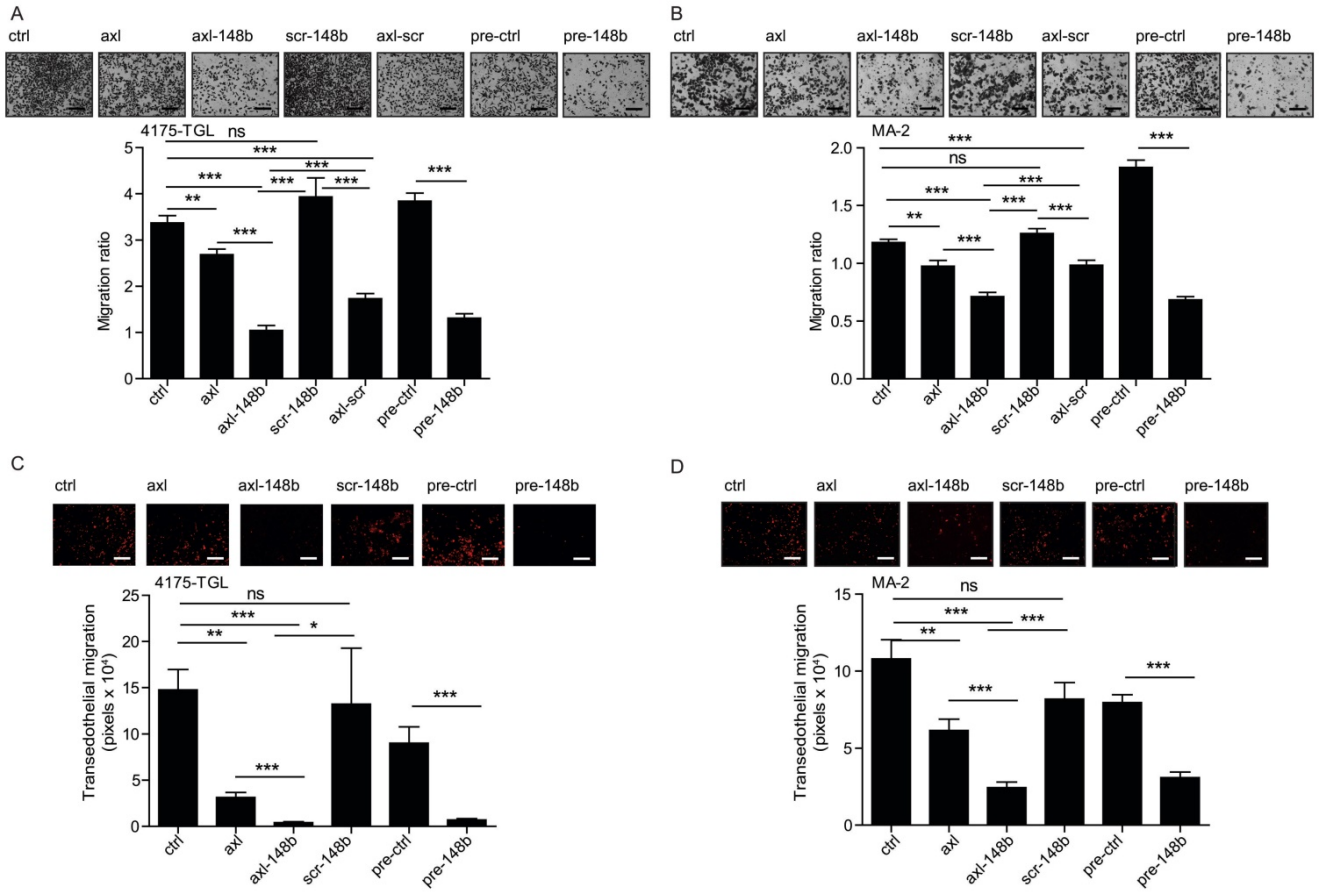
** Axl-148b conjugate inhibits tumor cell movement.** (A-D) Transwell migration assays were used to evaluate migration (A-B) or transendothelial migration through a HUVECs monolayer on top of a porous membrane (C-D) for the AXL^+^ 4175-TGL or MA-2 tumor cell lines left untreated (controls=ctrl) or treated with 400 nmol/L of axl, axl-148b, scr-148b or axl-scr aptamers. Alternatively, cells were transfected with 75 nmol/L of miR-148 precursor (pre-148b) or its negative control (pre-ctrl). Top: representative pictures of migrated cells. Bottom: result quantitations expressed as ratio of mean±SEM of the area covered by migrated versus plated tumor cells (A-B) or as mean±SEM of the area (pixels) covered by migrated cells (C-D). At least 2 independent experiments (with triplicates) were performed and representative results are shown. scr= scramble; ns = not significant; * p < 0.05, ** p < 0.01, *** p < 0.001; SEM = Standard Error of Mean; scale bar = 50 µm (A-D).

With the goal to evaluate the effects of axl-148b on metastatic traits, 4175-TGL and SKBR3 breast cancer, MA-2 melanoma or A549 lung cancer cells, were left untreated (ctrl) or treated with GL21.T, axl-148b, scr-148b or GL21.T aptamer linked to a scrambled miRNA sequence (axl-scr). Alternatively, cells were transfected with miR-148b precursors (pre-148b) or controls (pre-ctrl). Migration, invasion through Matrigel, transendothelial migration through a HUVECs monolayer and proliferation or viability assays were performed on treated cells. As shown in Figure 1A-D and S3A-D, an inhibitory effect on cell motility was observed for all AXL^+^, but not SKBR3 AXL^-^ cells, treated with GL21.T aptamer or axl-conjugates, as compared to control (ctrl or scr-148b) cells. Interestingly, a stronger effect was observed when axl-148b conjugates were used, indicating a combined functional action of GL21.T aptamer and miR-148b. A similar effect was observed for pre-148b transfected cells compared to pre-ctrl for both AXL^+^ or AXL^-^ cells. Observed data suggest that the biological effects of miR-148b following axl-148b conjugate administration depend on GL21.T-mediated delivery and, therefore, they are specific for AXL expressing cells. No effect on proliferation or viability was observed when cells were treated with GL21.T, axl-148b or scr-148b molecules or transfected with pre-148b compared to controls (ctrl or pre-ctrl), as in (Figure S4A-H), thus indicating an effect of miR-148b on cell movement only.

### Axl-148b conjugate affects miR-148b direct targets in tumor cells

**Figure 2:**
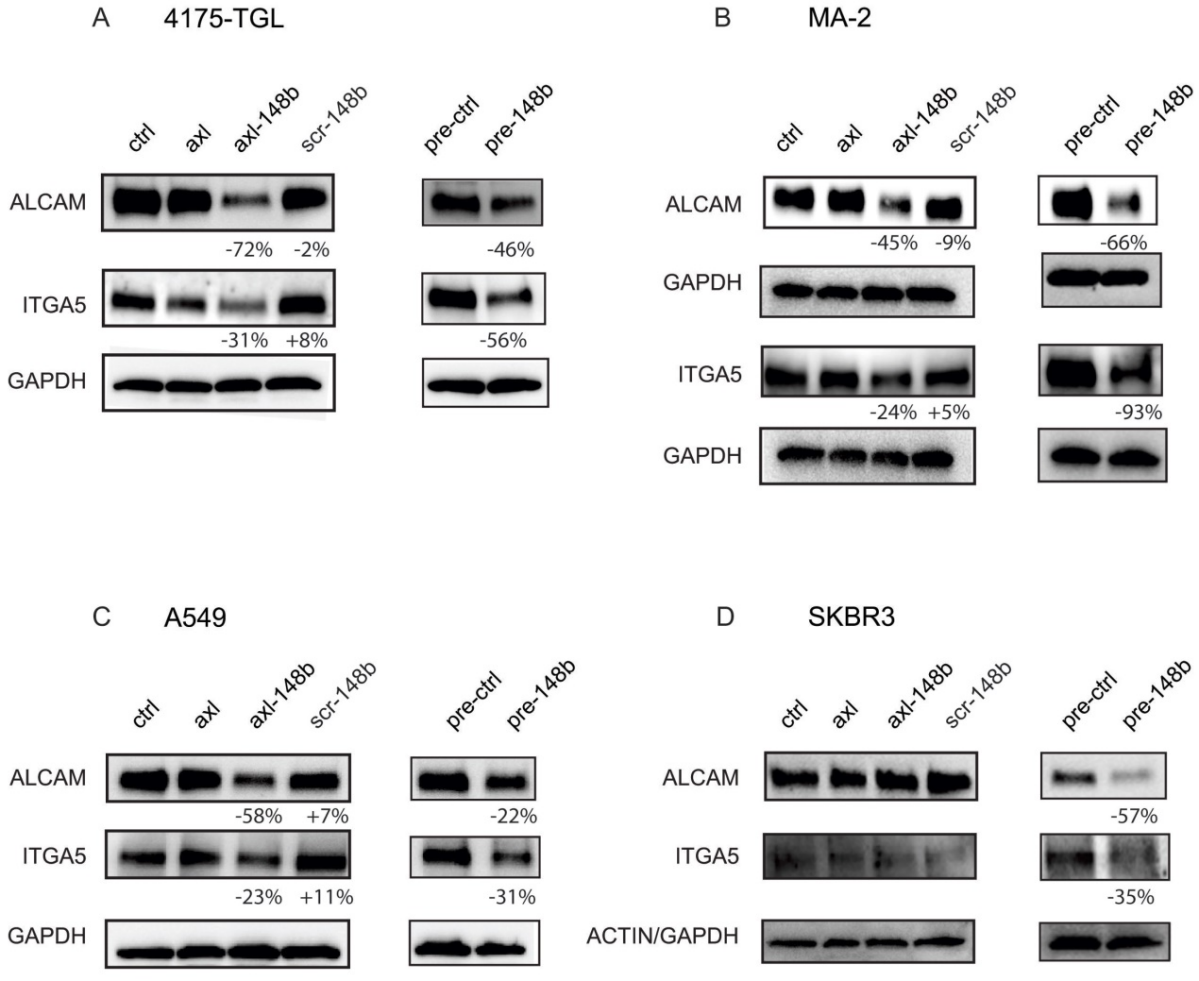
** Axl-148b conjugate affects ALCAM and ITGA5 expression in tumor cells.** (A-D) Western blot analysis of ALCAM and ITGA5 protein expression for the indicated AXL^+^ (A-C) or AXL^-^ (D) tumor cells left untreated (controls=ctrl) or treated with 400 nmol/L of axl, axl-148b or scr-148b aptamers. Alternatively, cells were transfected with 75 nmol/L of miR-148b precursors (pre-148b) or its negative control (pre-control). Filters were first stained for ALCAM, stripped and reprobed with anti-ITGA5 antibodies. Protein modulations were calculated relative to axl or controls (pre-ctrl), normalized on loading controls (GAPDH or actin) and expressed as percentages (%). At least 2 independent experiments were performed and representative results are shown. scr= scramble.

To explore the molecular mechanism underlying the effects of axl-148b chimeric molecules on metastatic traits, we evaluated the expression of ALCAM and ITGA5, two miR-148b-3p direct targets based on TargetScan 6.2 (http://www.targetscan.org/) and miRDB (http://mirdb.org/index.html) prediction algorithms, able to coordinate tumor cell extravasation 7, 8. We, therefore, treated 4175-TGL and SKBR3 breast cancer, MA-2 melanoma or A549 lung cancer cells, with GL21.T, axl-148b or scr-148b molecules and evaluated ALCAM and ITGA5 protein expression levels compared to cells transfected with miR-148b precursors (pre-148b) or controls (pre-ctrl). As shown in Figure 2A-D, ALCAM and ITGA5 expression was reduced in AXL^+^, but not AXL^-^ cells treated with axl-148b. Instead, all cells showed reduced levels of these two adhesion molecules when transfected with pre-148b, but not with pre-ctrl. In some cases, ALCAM and ITGA5 also resulted slightly modulated by GL21.T aptamer treatments, possibly because of a cross-talk between these transmembrane proteins and AXL. In summary, our results suggest that axl-148b chimeric conjugate acts similarly to pre-148b in the coordination of the molecular pathways involved in cell dissemination.

### Axl-148b conjugate affects mammosphere number and size

**Figure 3:**
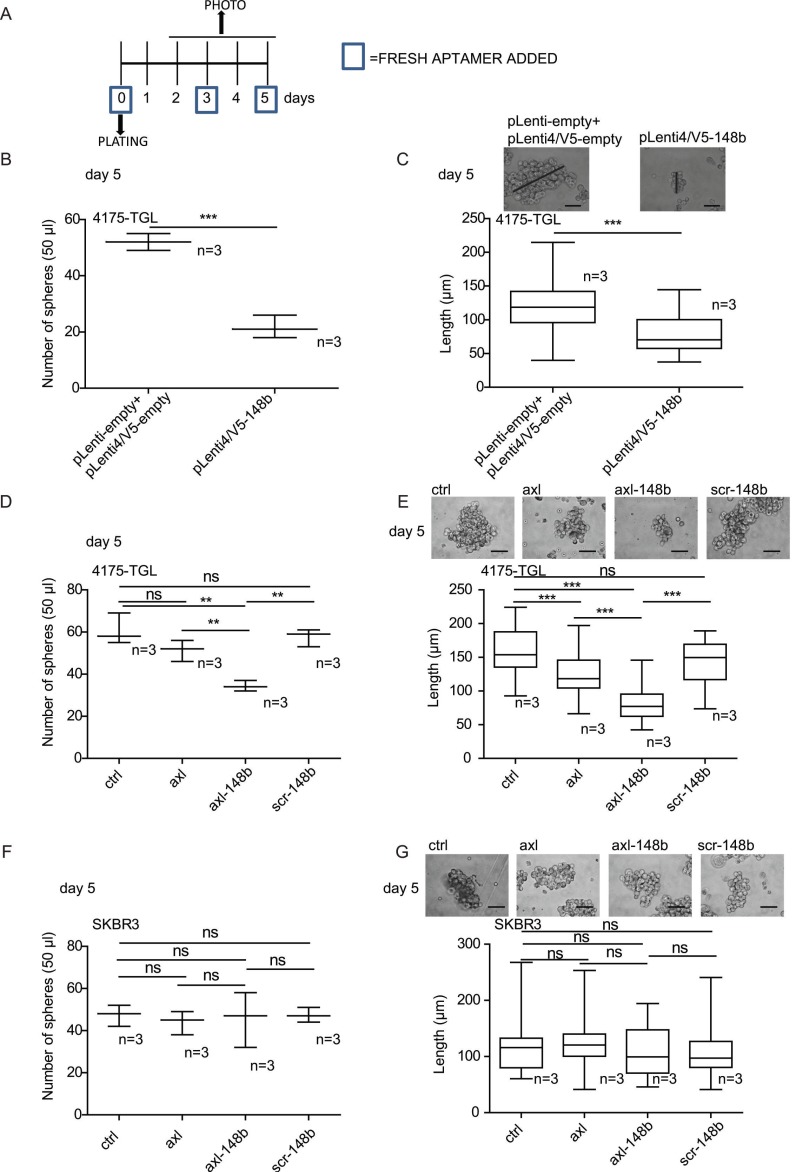
** Axl-148b conjugate inhibits formation and growth of mammospheres derived from AXL^+^ but not AXL^-^ tumor cells.** (A) Experimental scheme related to mammosphere assays referring to 4175-TGL or SKBR3 breast cancer cell lines. Cells were plated and grown in suspension for 5 days and treated with 200/400 nmol/L of axl, axl-148b or scr-148b aptamers at day 0, 3, 5, as indicated (numbers with squares). (B-C) Control (pLenti-empty + pLenti4/V5-empty) or miR-148b-overexpressing (pLenti4/V5-148b) 4175-TGL breast cancer cells were put in culture (day 0, suspension) and formation and size of spheres evaluated at day 5. (D-G) Formation and growth of spheres derived from 4175-TGL or SKBR3 breast cancer cells at day 5, treated as in (A). (B, D, F) Box-and-whisker plots for number of 4175-TGL or SKBR3 derived-mammospheres in 50 µl of culture volume at day 5 shown as mean±SEM. (C, E, G) Top: representative images of 4175-TGL or SKBR3 derived-mammospheres at day 5. Bottom: box-and-whisker plots of sphere size, shown as mean±SEM of sphere length (µm); black lines correspond to measurements for size. (B-G) 2 or 3 independent experiments (with triplicates) were performed and representative results are shown. scr= scramble; ns = not significant; * p < 0.05, ** p < 0.01, *** p < 0.001; SEM = Standard Error of Mean; scale bar = 25 µm (C, E, G).

Since Cancer Stem Cells (CSCs) are responsible for the metastatic spread 26, we evaluated the influence of axl-148b conjugate on the development of 3D mammospheres, derived from 4175-TGL and SKBR3 breast cancer cells. For this purpose, single cells were plated at day 0, left untreated (controls=ctrl) or treated with GL21.T, axl-148b or scr-148 molecules at day 0, 3 and 5 and mammospheres analyzed at day 5 (Figure 3A). Alternatively, single cells were plated at day 0 and derived mammospheres dissociated at day 5, re-plated and left untreated (controls=ctrl) or treated with GL21.T aptamer or axl-148b chimera at day 5, 8 or 10; mammospheres were then analyzed at day 12 (Figure S5A). For all experiments, miR-148b levels resulted increased in AXL^+^ 4175-TGL, but not in AXL^-^ SKBR3 breast cancer cells following treatments with axl-148b conjugates compared to ctrl or GL21.T-treated cells, as measured by qRT-PCR analysis (Figure S5B and not shown). In parallel, 4175-TGL cells overexpressing miR-148b (pLenti4/V5-148b) and empty controls (pLenti-empty+pLenti4/5V-empty) were also plated at day 0 and mammospheres analyzed at day 5. Here, we observed that pLenti4/V5-148b expressing cells generated a reduced number of spheres of a smaller size compared to pLenti-empty+pLenti4/5V-empty controls (Figure 3B-C). Similarly, when number or size of spheres or percentage (%) of PKH26 positive cells were evaluated in 4175-TGL-derived mammospheres at day 5 (Figure 3D-E) or at day 12 (Figure S5C-E), following the indicated treatments, a strong reduction in sphere formation was observed when cells were treated with axl-148b conjugates compared to ctrl, GL21.T or scr-148b cells. Instead, when axl-148b conjugate was used to treat AXL^-^ SKBR3-derived mammospheres, no effect on number or size of mammospheres was detected (Figure 3F-G). In summary, our data show that axl-148b influences mammosphere formation and growth in an AXL-dependent manner, suggesting the possibility to use this conjugate to block tumor cell stemness and subsequent metastatization.

### Axl-148b conjugate blocks tumor cell dissemination

**Figure 4:**
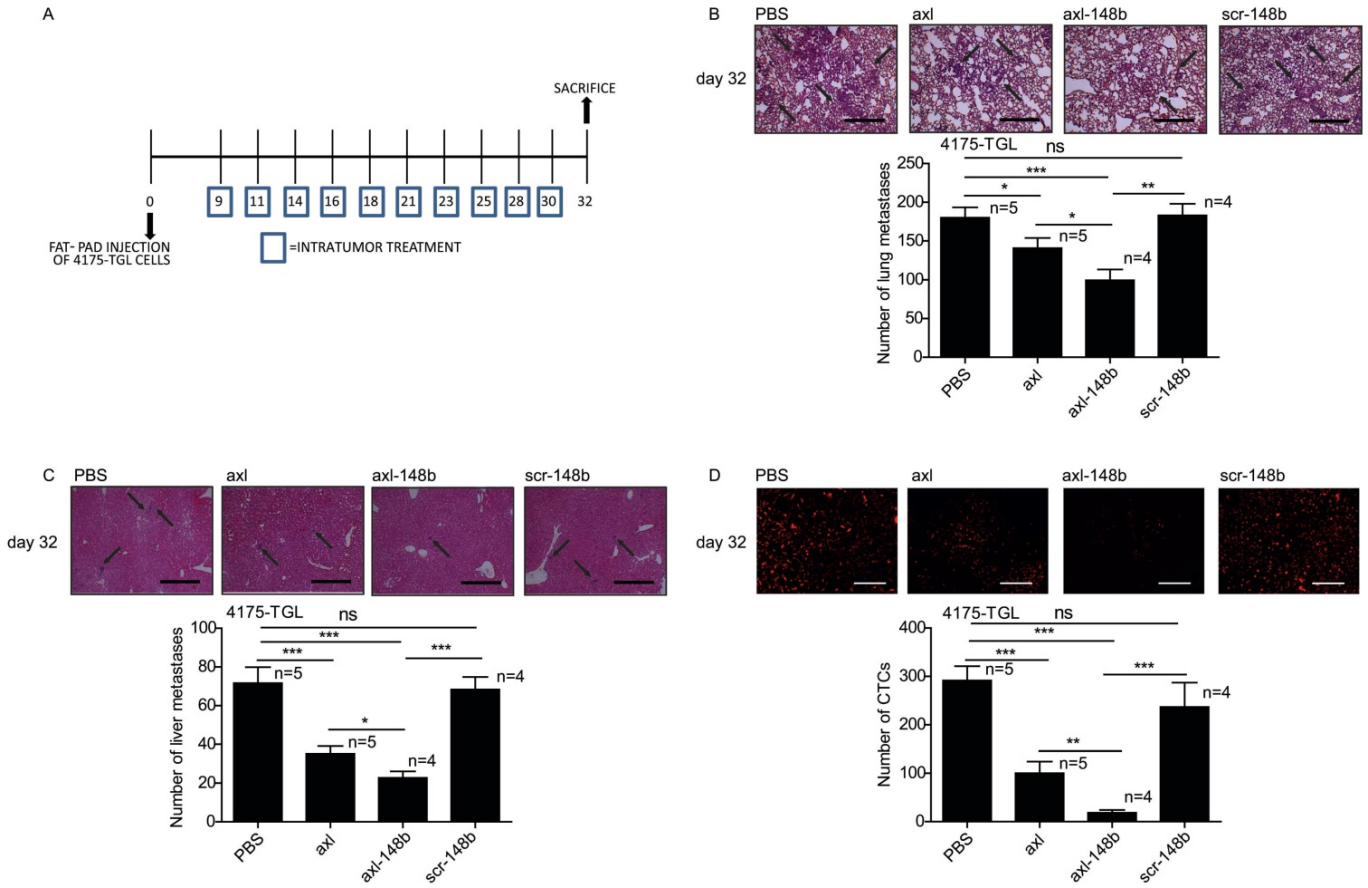
** Axl-148b conjugate prevents breast tumor dissemination in mice.** (A) Scheme of the experiment: Red fluorescent (RFP-expressing) 4175-TGL cells were injected into the mammary gland fat pad of NOD/SCID/IL2R null mice and PBS or axl, axl-148b or scr-148b aptamers were administered into the tumors starting at day 9 post-injection, when masses were palpable, (3 treatments/week, 300 pmol in 100 µl, 10 injections in total, as indicated) and lung/liver metastases and CTCs were evaluated 32 days post-tumor-cell injections. (B-D) Total number of lung/liver metastases or fluorescent CTCs are presented as mean±SEM for the indicated number (n) of mice. Representative images of lung/liver metastases or fluorescent CTCs are also shown. Arrows point to metastases. CTCs = Circulating Tumor Cells. scr= scramble. * p< 0.05, ** p< 0.01, *** p < 0.001. SEM = Standard Error of Mean. Scale bar=800 µm (B-C) or 50 µm (D).

**Figure 5:**
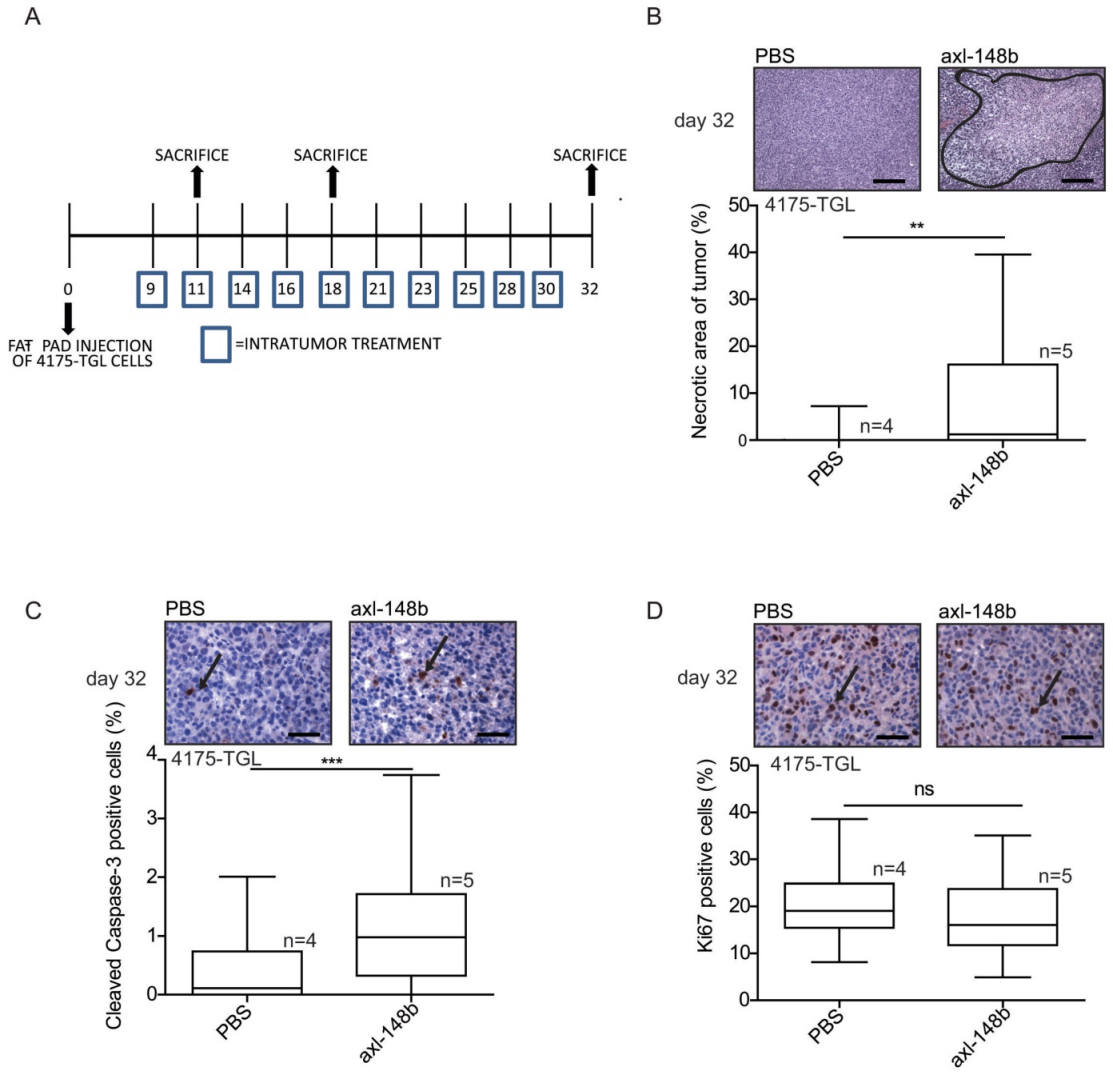
** Axl-148b conjugate promotes necrosis and apoptosis in breast cancer xenotransplants.** (A) Scheme of the experiment: Red fluorescent (RFP-expressing) 4175-TGL cells were injected into the mammary gland fat pad of NOD/SCID/IL2R null mice and PBS or axl-148b aptamers were administered into the tumors starting at day 9 post-injection, when masses were palpable, (3 treatments/week, 300 pmol in 100 µl, 10 injections in total, as indicated) and lung metastases were evaluated 11, 18 or 32 days post-tumor-cell injections. (B) FFPE sections of primary tumors were stained with H&E and necrotic areas evaluated: representative images are shown on top of box-and-whisker plots presenting the percentage (%) of necrotic (delimited) versus total areas shown as mean±SEM for the indicated number (n) of mice. (C-D) Primary tumors were stained with Cleaved Caspase-3 (C) or Ki-67 (D) antibodies by IHC and nuclei were counterstained with Hematoxylin (blue): representative images are shown on top of box-and-whisker plots presenting the percentage (%) of positive versus total cells shown as mean±SEM for the indicated number (n) of mice (10 fields/each mouse were evaluated). IHC = immunohistochemistry; H&E = Hematoxilyin & Eosin; FFPE: Formalin-Fixed, Paraffin Embedded; arrows point to positive cells; ns = not significant; * p< 0.05, ** p< 0.01, *** p < 0.001; SEM = Standard Error of Mean; scale bar = 100 µm (B) or 25 µm (C-D).

**Figure 6:**
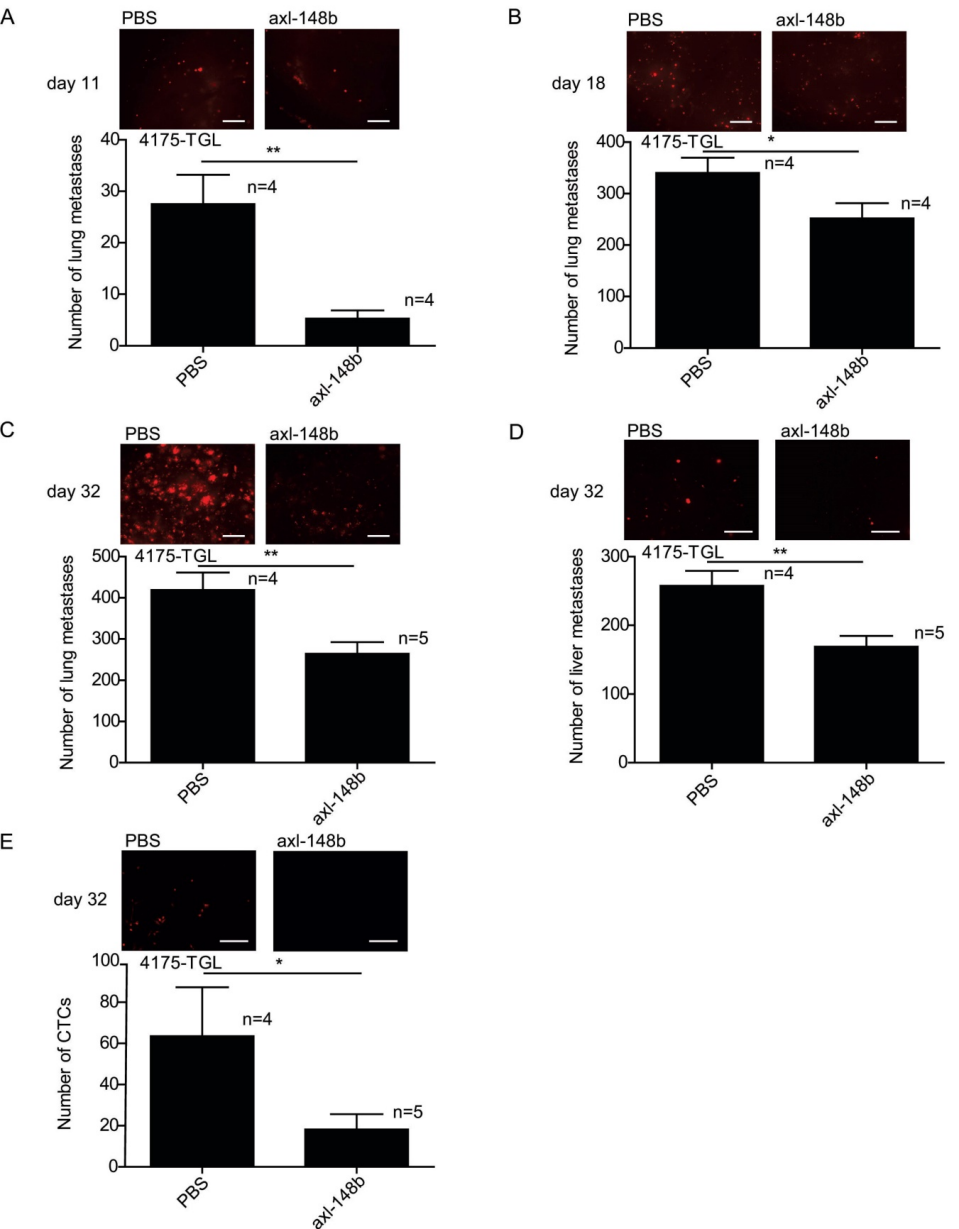
** Axl-148b conjugate prevents breast tumor dissemination in mice in a time-course model.** Refer to Figure 5A for scheme of the experiment. (A-E) Total number of lung/liver fluorescent metastases or CTCs were evaluated at 11 or 18 or 32 days post-tumor-cell injections and are presented as mean±SEM for the indicated number (n) of mice. Representative images are also shown. CTCs = Circulating Tumor Cells; * p< 0.05, ** p< 0.01, *** p < 0.001; SEM = Standard Error of Mean; scale bar=800 µm (A-D) or 50 µm (E).

In order to evaluate the efficacy of axl-148b chimera on primary tumors and metastatic dissemination in mice, tRFP-positive 4175-TGL breast cancer cells were injected into the mammary gland fat-pad of NSG immunocompromised mice and GL21.T, axl-148b, scr-148b or PBS (control) were administered into the primary mass, 3 times/week, starting when tumors were palpable (day 9), as in Figure 4A. Metastasis formation as well as Circulating Tumor Cells (CTCs) were analyzed at day 32 post-injection. Here, a significant reduction of lung or liver metastases and CTC number was observed in GL21.T or axl-148b treated mice compared to controls (PBS or scr-148b) at day 32 (Figure 4B-D). Importantly, a pronounced reduction of metastases and CTCs was detected in axl-148b treated mice compared to GL21.T injected animals (Figure 4B-D) while scr-148b injected mice behaved as PBS treated ones. In other experiments, tRFP-positive 4175-TGL breast cancer or MA-2 melanoma cells were injected respectively into the mammary gland fat-pad or in the flank (subcutaneously) of NSG immunocompromised mice and axl-148b or PBS (control) were delivered to mice (3 times/week) starting when tumors were palpable (day 9 for 4175-TGL or 12 for MA-2), as shown in Figure 5A and S6A. Primary tumor characteristics, metastasis formation as well as CTCs were analyzed at day 11, 18 or 32 post-injection (Figure 5, 6, S6, S7). No consistent reduction of tumor weight was observed for all conjugate-treated primary tumors compared to controls at day 32 (data not shown). However, H&E stainings showed increased tumor necrosis (Figure 5B and S6B), while IHC analysis for Cleaved Caspase-3 and Ki67 revealed increased apoptosis, but no alteration in proliferation (Figure 5C-D and S6C-D). More importantly, a significant reduction of lung metastases formation was observed in axl-148b conjugate-treated mice compared to controls at day 11, 18 and 32 for 4175-TGL (Figure 6A-C). In the injected animals a pronounced reduction of liver metastases or CTCs was also detected at day 32 (Figure 6D-E and S6E). Analysis on AXL levels revealed that 62% of 4175-TGL cells in culture express AXL (Figure S7A). IHC analyses on primary tumors showed no differences in AXL expression at day 11 and an average of 40% AXL positive cells were evidenced in all tumors (Figure S7B). Instead, a significative reduction of AXL^+^ cells was found at day 18 and 32 in axl-148b injected tumors compared to controls (Figure S7C-D). Surprisingly, no differences in AXL levels were detected in metastases at day 32 (Figure S7E). Interestingly, axl-148b levels increased in primary tumors treated with the chimera at day 11, but not at day 18 or 22, probably because of increased loss of AXL^+^ cells at later time points (Fig S7F). Relevantly, following axl-148b conjugate administration, no toxicity was evidenced in the animals compared to controls. In fact, no morphology (analyzed by H&E staining) or weight alterations were detected for liver, spleen and kidneys (Figure S8A-C), at the end of the experiment as in Figure 5A. Together, these results suggest that axl-148b conjugate is a promising pre-clinical therapeutic tool able to affect primary tumor structure and cell dissemination in mice, with no signs of toxicity and a good potential for the translation to the clinic.

## Discussion

We previously reported that the simultaneous inhibition of the pro-metastatic miR-214 and the overexpression of the anti-metastatic miR-148b strongly affected metastasis formation in mice by reducing tumor cell extravasation 8. With the goal to evaluate the therapeutic potential of miR-148b, we generated a chimeric molecule for the specific delivery of miR-148b to tumor cells. Precisely, we conjugated a well-established aptamer against AXL (GL21.T), a tyrosine kinase receptor overexpressed in many tumor cells, with miR-148b and generated an axl-148b conjugate able to increase miR-148b expression specifically in AXL^+^, but not AXL^-^ cells and to reduce cell motility and mammosphere formation *in vitro*. More importantly, following axl-148b conjugate administration into mouse xenotransplants, increased tumor necrosis and apoptosis were observed in the primary masses, together with a strong reduction of CTCs and metastasis formation in distant organs.

The chimeric axl-148b molecule used in our investigation resulted highly specific for AXL^+^ cells, similarly to what observed here or previously for GL21.T 18-20, 25. In fact, treatments with axl-148b conjugate allowed an efficient increase of miR-148b in AXL^+^ but not in AXL^-^ cells. Therefore, suggesting that the conjugation of miR-148b to GL21.T did not alter the specific AXL binding, internalization and signaling inhibition in AXL^+^ cells 19, 20, 25 and, at the same time, it guaranteed cytoplasmic Dicer processing, leading to the production of mature miR-148b. Similar results can be found in the literature for other conjugated miRNAs or anti-miRNAs, such as the tumor suppressor small non-coding RNAs let-7g 19, miR-212 20 and miR-137 21 or the anti-miR-222, a sequence able to inhibit the pro-tumorigenic miR-222 25, or siRNAs 27. Our data also confirm that the generated miR-148b is mature and fully functional. In fact, two well-established miR-148b direct targets, the adhesion molecules ALCAM and ITGA5, known to be involved in migration and extravasation of tumor cells 7, 8, appeared down-regulated following cell treatments with axl-148b conjugate, similarly to cells engineered for the overexpression of miR-148b. More importantly, the generated axl-148b molecule showed strong inhibitory effects in tumor progression in *in vitro* and *in vivo* experiments.

*In vitro* axl-148b chimera was able to decrease cell motility or invasion for breast cancer and melanoma cells, in a stronger and additive manner compared to GL21.T aptamer alone 18, and similarly to what evidenced for miR-148b overexpressing cells 7, 8. Instead, no effect on breast cancer and melanoma cell growth or viability was observed for 2D plated cells, in agreement with our previous studies in which cells were treated with GL21.T 18 or previously engineered for miR-148b expression 7, 8. Considering the importance of CSCs in tumor progression 26 and the well-known involvement of AXL in the control of breast cancer 28 and cutaneous tumor 29 stemness, we evaluated the effects of axl-148b conjugate on a 3D model of breast cancer mammospheres 30-32. Relevantly, an inhibition of mammosphere formation and growth was observed when AXL^+^, but not AXL^-^ cells were treated with GL21.T aptamer or axl-148b conjugate. However, the impairment of sphere formation/growth was stronger when axl-148b chimera was used compared to treatments with GL21.T, while it was similar to what observed for miR-148b overexpressing cells, therefore suggesting a negative effect of miR-148b on breast CSCs. Comparably, a similar effect has been previously observed for miR-148b in HepatoCellular Carcinoma (HCC) stemness 33.

More relevantly, *in vivo*, axl-148b conjugate was able to alter primary tumor structures and to impair dissemination of metastatic cells when administered into mouse xenotransplants. Precisely, increased necrosis and apoptosis were observed in AXL^+^-treated tumors compared to controls. Of great importance, intratumor treatments of GL21.T or axl-148b led to a strong reduction in CTCs and metastasis formation in distant organs (lung and liver) and axl-148b effects were stronger than GL21.T alone, as expected by the anti-metastatic potential of miR-148b. In line with this, a modified GL21.T aptamer was shown to inhibit ovarian cancer dissemination 34, while our previous *in vivo* studies revealed a strong impairment of metastasis formation for melanoma or breast cancer cells overexpressing miR-148b 7, 8.

Furthermore, we demonstrated axl-148b ability to reduce the percentage of AXL^+^ cells in the tumor at 18 and 32 days. Considering that toxicity is a major hurdle for the *in vivo* delivery of therapeutics, we evaluated the possible side effects of axl-148b conjugate in treated mice by analyzing morphology and weight of the main internal organs, such as liver, spleen and kidneys and no alterations have been evidenced. Furthermore, no increase in inflammatory cytokines was previously observed in liver and spleen of animals treated with GL21.T aptamers 19, 25, therefore suggesting the potential transfer of our conjugates to the clinics. However, the low *in vitro* stability (up to 8 hours) of our 2'F-Py and 2'OMe-Pu modified conjugate and of other similarly stabilized GL21.T-chimeric aptamers is an issue for the transfer to the clinics 25. Nevertheless, recent data show that it is possible to improve conjugate stability *in vitro* (up to 24 hours) by inserting monothiophosphate modifications 34, an intervention that we can apply to our aptamer. Moreover, we also plan to reduce renal clearance by embedding our conjugates in biotin, cholesterol or polyethylene glycol (PEG), as done for similar molecules in 35. Therefore, with these interventions we should be able to obtain a conjugate that can be effective in *in vivo* metastasis treatment following systemic delivery, making the molecule transferable to the clinics.

## Conclusions

Our work provides evidence that axl-148b conjugate can be specifically delivered to AXL^+^ but not AXL^-^ cells, therefore leading to increased miR-148b levels and downregulation of its targets, ALCAM and ITGA5. As a consequence, treated cells showed decreased motility and ability to form spheres *in vitro,* while *in vivo,* pronounced necrosis and apoptosis were observed in treated xenotransplants, associated with decreased tumor cell dissemination and metastasis formation in distant organs with no sign of toxicity. Thus, providing a proof of concept for a targeted therapy of highly malignant melanomas and breast cancers.

## Supplementary Material

Supplementary figures and table.

## Abbreviations

CLL: Chronic Lymphocytic Leukemia
CSCs: Cancer Stem Cells
CTCs: Circulating Tumor Cells
FFPE: Formalin Fixed Paraffin Embedded
HCC: HepatoCellular Carcinoma
H&E: Hematoxylin & Eosin
HUVECs: Human Umbilical Vein Endothelial Cells
IHC: ImmunoHistoChemistry
miRNA: microRNA
PEG: polyethylene glycol
SD: Standard Deviation
SEM: Standard Error of Mean
WB: Western Blot

## References

[1] Valastyan S, Weinberg RA (2011). Tumor metastasis: molecular insights and evolving paradigms. Cell.

[2] Calin GA, Dumitru CD, Shimizu M, Bichi R, Zupo S, Noch E (2002). Frequent deletions and down-regulation of micro- RNA genes miR15 and miR16 at 13q14 in chronic lymphocytic leukemia. Proc Natl Acad Sci U S A.

[3] Mueller DW, Rehli M, Bosserhoff AK (2009). miRNA expression profiling in melanocytes and melanoma cell lines reveals miRNAs associated with formation and progression of malignant melanoma. J Invest Dermatol.

[4] Penna E, Orso F, Cimino D, Tenaglia E, Lembo A, Quaglino E (2011). microRNA-214 contributes to melanoma tumour progression through suppression of TFAP2C. EMBO J.

[5] Penna E, Orso F, Cimino D, Vercellino I, Grassi E, Quaglino E (2013). miR-214 coordinates melanoma progression by upregulating ALCAM through TFAP2 and miR-148b downmodulation. Cancer Res.

[6] Le Quesne J, Caldas C (2010). Micro-RNAs and breast cancer. Mol Oncol.

[7] Cimino D, De Pitta C, Orso F, Zampini M, Casara S, Penna E (2013). miR148b is a major coordinator of breast cancer progression in a relapse-associated microRNA signature by targeting ITGA5, ROCK1, PIK3CA, NRAS, and CSF1. FASEB J.

[8] Orso F, Quirico L, Virga F, Penna E, Dettori D, Cimino D (2016). miR-214 and miR-148b Targeting Inhibits Dissemination of Melanoma and Breast Cancer. Cancer Res.

[9] van der Ree MH, de Vree JM, Stelma F, Willemse S, van der Valk M, Rietdijk S (2017). Safety, tolerability, and antiviral effect of RG-101 in patients with chronic hepatitis C: a phase 1B, double-blind, randomised controlled trial. Lancet.

[10] Beg MS, Brenner AJ, Sachdev J, Borad M, Kang YK, Stoudemire J (2017). Phase I study of MRX34, a liposomal miR-34a mimic, administered twice weekly in patients with advanced solid tumors. Invest New Drugs.

[11] Dettori D, Orso F, Penna E, Baruffaldi D, Brundu S, Maione F (2018). Therapeutic Silencing of miR-214 Inhibits Tumor Progression in Multiple Mouse Models.

[12] Zhou J, Rossi JJ (2014). Cell-type-specific, Aptamer-functionalized Agents for Targeted Disease Therapy. Mol Ther Nucleic Acids.

[13] Tan L, Neoh KG, Kang ET, Choe WS, Su X (2011). PEGylated anti-MUC1 aptamer-doxorubicin complex for targeted drug delivery to MCF7 breast cancer cells. Macromol Biosci.

[14] Ray P, Cheek MA, Sharaf ML, Li N, Ellington AD, Sullenger BA (2012). Aptamer-mediated delivery of chemotherapy to pancreatic cancer cells. Nucleic Acid Ther.

[15] McNamara JO 2nd, Andrechek ER, Wang Y, Viles KD, Rempel RE, Gilboa E (2006). Cell type-specific delivery of siRNAs with aptamer-siRNA chimeras. Nat Biotechnol.

[16] Chu TC, Twu KY, Ellington AD, Levy M (2006). Aptamer mediated siRNA delivery. Nucleic Acids Res.

[17] Dai F, Zhang Y, Zhu X, Shan N, Chen Y (2012). Anticancer role of MUC1 aptamer-miR-29b chimera in epithelial ovarian carcinoma cells through regulation of PTEN methylation. Target Oncol.

[18] Cerchia L, Esposito CL, Camorani S, Rienzo A, Stasio L, Insabato L (2012). Targeting Axl with an high-affinity inhibitory aptamer. Mol Ther.

[19] Esposito CL, Cerchia L, Catuogno S, De Vita G, Dassie JP, Santamaria G (2014). Multifunctional aptamer-miRNA conjugates for targeted cancer therapy. Mol Ther.

[20] Iaboni M, Russo V, Fontanella R, Roscigno G, Fiore D, Donnarumma E (2016). Aptamer-miRNA-212 Conjugate Sensitizes NSCLC Cells to TRAIL. Mol Ther Nucleic Acids.

[21] Esposito CL, Nuzzo S, Kumar SA, Rienzo A, Lawrence CL, Pallini R (2016). A combined microRNA-based targeted therapeutic approach to eradicate glioblastoma stem-like cells. J Control Release.

[22] Xu L, Shen SS, Hoshida Y, Subramanian A, Ross K, Brunet JP (2008). Gene expression changes in an animal melanoma model correlate with aggressiveness of human melanoma metastases. Mol Cancer Res.

[23] Minn AJ, Gupta GP, Siegel PM, Bos PD, Shu W, Giri DD (2005). Genes that mediate breast cancer metastasis to lung. Nature.

[24] Amarzguioui M, Lundberg P, Cantin E, Hagstrom J, Behlke MA, Rossi JJ (2006). Rational design and in vitro and in vivo delivery of Dicer substrate siRNA. Nat Protoc.

[25] Catuogno S, Rienzo A, Di Vito A, Esposito CL, de Franciscis V (2015). Selective delivery of therapeutic single strand antimiRs by aptamer-based conjugates. J Control Release.

[26] Shiozawa Y, Nie B, Pienta KJ, Morgan TM, Taichman RS (2013). Cancer stem cells and their role in metastasis. Pharmacol Ther.

[27] Zhou J, Li H, Li S, Zaia J, Rossi JJ (2008). Novel dual inhibitory function aptamer-siRNA delivery system for HIV-1 therapy. Mol Ther.

[28] Chang TY, Chen HA, Chiu CF, Chang YW, Kuo TC, Tseng PC (2016). Dicer Elicits Paclitaxel Chemosensitization and Suppresses Cancer Stemness in Breast Cancer by Repressing AXL. Cancer Res.

[29] Cichon MA, Szentpetery Z, Caley MP, Papadakis ES, Mackenzie IC, Brennan CH (2014). The receptor tyrosine kinase Axl regulates cell-cell adhesion and stemness in cutaneous squamous cell carcinoma. Oncogene.

[30] Kreso A, Dick JE (2014). Evolution of the cancer stem cell model. Cell Stem Cell.

[31] Zhang L, Wang H, Li C, Zhao Y, Wu L, Du X (2017). VEGF-A/Neuropilin 1 Pathway Confers Cancer Stemness via Activating Wnt/beta-Catenin Axis in Breast Cancer Cells. Cell Physiol Biochem.

[32] Liu W, Wu T, Dong X, Zeng YA (2017). Neuropilin-1 is upregulated by Wnt/beta-catenin signaling and is important for mammary stem cells. Sci Rep.

[33] Liu Q, Xu Y, Wei S, Gao W, Chen L, Zhou T (2015). miRNA-148b suppresses hepatic cancer stem cell by targeting neuropilin-1.

[34] Kanlikilicer P, Ozpolat B, Aslan B, Bayraktar R, Gurbuz N, Rodriguez-Aguayo C (2017). Therapeutic Targeting of AXL Receptor Tyrosine Kinase Inhibits Tumor Growth and Intraperitoneal Metastasis in Ovarian Cancer Models. Mol Ther Nucleic Acids.

[35] Ni S, Yao H, Wang L, Lu J, Jiang F, Lu A (2017). Chemical Modifications of Nucleic Acid Aptamers for Therapeutic Purposes.

