# Supplementary material for: Axl-148b chimeric aptamers inhibit breast cancer and melanoma progression
Source: Int J Biol Sci. 2020 Feb 10;16(7):1238–51. doi: 10.7150/ijbs.39768 (PMC7053324; doi:10.7150/ijbs.39768)
Supplement: Supplementary file 1 — Supplementary figures and table. [file ijbsv16p1238s1.pdf]

SUPPLEMENTARY TABLE AND FIGURE LEGENDS

Table S1- miR-148b-3p or U44 Cts ± SD and ΔCt ± SD for the different cell lines referring to Fig. S2. SD= Standard Deviation. ΔCt=miR-148b-3p-U44.

Figure S1 – Axl-148b structure, annealing, stability and binding to AXL. (A) Schematic representation of the axl-148b aptamer conjugate. (B) Representative image of non-denaturing polyacrylamide gel electrophoresis showing 3P or 5P miR-148b oligonucleotide sequences and axl aptamers or axl-148b conjugates, stained with ethidium-bromide. Proper annealing was checked for every aptamer preparation. (C) Representative image of non-denaturing polyacrylamide gel electrophoresis showing axl-148b conjugate (4 μM) stability in presence of 80% human serum for the indicated times (hours=h), stained with ethidium-bromide. (B-C) Referral= 1kb ladder DNA. (D) Binding of 400 nmol/L of axl or axl-148b conjugates on A549 cells measured by qRT-PCR after 30 min of incubation.

Figure S2 – AXL, miR-148b or let-7g expression in tumor cells treated or not with axl, axl-148b, scr-148b or axl-let-7g molecules. (A) qRT-PCR analysis of AXL mRNA levels in AXL^+ A549 lung cancer cells, MA-2, MC-1 melanoma cells and MDAMB231, 4175-TGL breast cancer cells or AXL^- SKBR3 breast cancer cells. Results are shown as fold changes (mean±SD) relative to A549 cells, normalized on GAPDH levels. Two experiments were performed and a representative one is shown. (B-G) qRT-PCR analysis for miR-148b-3p (indicated as miR-148b) (B-F) or let-7g (G) expression for the indicated cell lines left untreated (controls = ctrl) or treated with 400 nmol/L of axl, axl-148b, scr-148b or axl-let7g aptamers. Alternatively, cells were transfected with 75 nmol/L of miR-148b precursor (pre-148b) or its negative control (pre-ctrl). Results are shown as fold changes (mean±SD) relative to controls (ctrl or pre-ctrl), normalized on U6 or U44 small nucleolar RNA levels. 2 experiments (with triplicates) were performed and a representative one is

shown. scr= scramble. ns = not significant; * $p < 0.05$, ** $p < 0.01$, *** $p < 0.001$. SD = standard deviation.

Figure S3 – Axl-148b conjugate inhibits tumor cell movement in AXL^{+}, but not in AXL^{-} tumor cells. (A-D) Transwell assays were used to evaluate invasion through matrigel (A-B) or migration (C-D) for AXL^{+} 4175-TGL, MA-2 and A549 or AXL^{-} SKBR3 cells left untreated (controls = ctrl) or treated with 400 nmol/L of axl, axl-148b or scr-148b aptamers as indicated. Alternatively, cells were transfected with 75 nmol/L of miR-148 precursor (pre-148b) or its negative control (pre-ctrl). Representative pictures of invaded/migrated cells are shown on top of histograms referring to the ratio of mean±SEM of the area covered by invaded/migrated versus plated tumor cells. At least 2 independent experiments (with triplicates) were performed and representative results are shown. scr= scramble. ns = not significant; * $p < 0.05$, ** $p < 0.01$, *** $p < 0.001$; SEM = Standard Error of Mean; scale bar = 50 µm (A-D).

Figure S4 – Axl-148b conjugate does not significantly affect tumor cell growth. (A-H) Proliferation (A-B, E-F) or cell viability (C-D, G-H) of 4175-TGL (A-D) or MA-2 (E-H), cells left untreated (controls=ctrl) or treated with 400 nmol/L of axl, axl-148b or scr-148b aptamers. Alternatively, cells were transfected with 75 nmol/L of miR-148b precursor (pre-148b) or its negative control (pre-ctrl). Results are indicated as mean±SD of the proliferation or cell viability ratio versus plated cells, measured by optical density at 0-96h. At least 2 independent experiments (with triplicates) were performed and representative results are shown. scr= scramble; SD = Standard Deviation.

Figure S5– Axl-148b conjugate inhibits formation and growth of mammospheres derived from AXL$^+$ tumor cells. (A) Experimental scheme related to mammosphere assays for 4175-TGL breast cancer cells plated and grown in suspension for 5 days with no treatment, dissociated at day 5, re-plated and treated with 200/400 nmol/L of axl or axl-148b aptamers at day 5, 8 and 10, as indicated (numbers with squares) and evaluated at day 12. **(**B) qRT-PCR analysis for miR-148b expression in 4175-TGL derived-mammospheres left untreated (controls = ctrl) or treated with 200/400 nmol/L of axl or axl-148b aptamers. Results are shown as fold changes (mean±SD of triplicates) relative to controls (ctrl), normalized on U44 small nucleolar RNA levels. (C) Box-and-whisker plots of mammosphere number is shown, referring to 50 µl volume. (D) Representative images of mammospheres are presented on top of box-and-whisker plots referring to sphere dimensions as mean±SEM of sphere length (µm); black lines correspond to length measurements. (E) FACS analysis was used to evaluated the percentage (%) of PKH26 positive cells (versus total) in 4175-TGL derived-mammospheres at day 12, following dye treatment at day 5, shown as mean±SEM in box-and-whisker plots. For all results, 2 experiments with triplicates were performed and representative ones are shown. ns = not significant; * $p < 0.05$, ** $p < 0.01$, *** $p < 0.001$; SEM = Standard Error of Mean; scale bar = 25 µm (D).

Figure S6- Axl-148b conjugate induces primary tumor necrosis/apoptosis and prevents melanoma dissemination in mice. (A) Scheme of the experiment: Red fluorescent (RFP-expressing) MA-2 cells were injected into the flank of NOD/SCID/IL2R null mice and PBS or axl-148b aptamers were administered into the tumor starting at day 12 post-injection when tumors were palpable, (3 treatments/week, 300 pmol in 100 µl, 9 injections in total, as indicated) CTCs or primary tumors growth characteristics were analyzed 32 days after tumor-cell injections. (B) FFPE sections of primary tumors were stained with H&E and necrotic areas evaluated: representative images are shown on top of box-and-whisker plots presenting the percentage (%) of necrotic (delimited) versus total areas shown as mean±SEM for the indicated number (n) of mice. (C-D)

Primary tumors were stained by IHC with Cleaved Caspase-3 (C) or Ki-67 (D) antibodies and nuclei were counterstained with Hematoxylin (blue): representative images are shown on top of box-and-whisker plots presenting the percentage (%) of positive versus total cells shown as mean±SEM for the indicated number (n) of mice (10 fields/each mouse were evaluated). (E) Evaluation of CTCs: representative images are presented on top of box-and-whisker plots representing the total number of RFP-positive cells obtained from blood (32 days post-injection), grown in culture for 7 days, shown as mean±SEM for the indicated number (n) of mice. FFPE: Formalin-Fixed, Paraffin Embedded; arrows point to positive cells; IHC = immunohistochemistry; H&E = Hematoxilyin & Eosin; CTCs = circulating tumor cells; * $p < 0.05$, ** $p < 0.01$, *** $p < 0.001$; SEM = Standard Error of Mean; scale bar = 100 µm (B), 25 µm (C-D), 50 µm (E),

Figure S7 - AXL expression in primary tumors and metastases following axl-148b conjugate administration. (A-E) 4175-TGL cells in culture (A), or in primary tumors (B-D) or metastases (E), following primary tumor treatments as in Figure 5A, were stained with anti-AXL antibody by IHC and nuclei were counterstained with Hematoxylin (blue): representative images are shown on top of box-and-whisker plots representing the percentage (%) of AXL positive versus total cells shown as mean±SEM for 5 or 10 fields/mouse. (F) qRT-PCR analysis for miR-148b expression in primary tumors treated as in Figure 5A. Results are shown as fold changes (mean±SD) relative to controls (PBS), normalized on U6 small nucleolar RNA levels. 2 experiments (with triplicates) were performed and a representative one is shown. IHC = immunohistochemistry; n=number of mice; ns = not significant; * $p < 0.05$, ** $p < 0.01$, *** $p < 0.001$; SEM = Standard Error of Mean; scale bar = 25 µm.

Figure S8 – Axl-148b conjugate is not toxic for mice. (A-C) Mice were injected with Red fluorescent (RFP-expressing) 4175-TGL cells and treated as described in Figure 5A. Liver (A), spleen (B) and kidneys (C) were weighted at day 32 (final point) and FFPE sections stained with

H&E. Representative H&E pictures (bar=100 or 200 μm) are shown on top of box-and-whiskers plots representing the mean±SEM of organ weights for the indicated number (n) of mice. ns = not significant; SEM= Standard Error of Mean; H&E = Hematoxilyn & Eosin; FFPE: Formalin-Fixed, Paraffin Embedded; mg = milligrams.

Table S1

**A549**	ctrl	axl	axl-148b	scr-148b	pre-ctrl	pre-148b
miR-148b-3p	31.616 ± 0.26	31.86 ± 0,11	25.602 ± 0.031	32.693 ± 0.243	33.555 ± 0.516	24.991 ± 0.079
U44	26.542 ± 0.06	27.51 ± 0.115	26.331 ± 0.145	28.492 ± 0.048	28.042 ± 0.058	27.446 ± 0.036
ΔCt	5.074 ± 0.267	4.35 ± 0.159	-0.729 ± 0.148	4.201 ± 0.248	5.5132 ± 0.519	-2.455± 0.087

**4175-TGL**	ctrl	axl	axl-148b	scr-148b	pre-ctrl	pre-148b
miR-148b-3p	33.536 ± 0.467	33.644 ± 0.676	29.656 ± 0.054	33.49 ± 0.257	33.697 ± 0.412	21.668 ± 0.033
U44	30.273 ± 0.14	30.338 ± 0.177	31.998 ± 0.18	30.501 ± 0.014	27.564 ± 0.186	26.898 ± 0.111
ΔCt	3.263 ± 0.487	3.306 ± 0.699	-2.342 ± 0.188	2.989 ± 0.257	6.133 ± 0.452	-5,23 ± 0.116

**MA-2**	ctrl	axl	axl-148b	scr-148b	pre-ctrl	pre-148b
miR-148b-3p	31.976 ± 0.032	32.376 ± 0.041	30.079 ± 0.11	32.013 ± 0.146	33.199 ± 0.166	24.929 ± 0.31
U44	24.994 ± 0.044	24.974 ± 0.039	25.691 ± 0.068	24.829 ± 0.013	25.178 ± 0.076	25.563 ± 0.056
ΔCt	6,982 ± 0.054	7.402 ± 0.056	4.388 ± 0.129	7.184 ± 0.146	8.021 ± 0.182	-0.634 ± 0.315

**SKBR3**	ctrl	axl	axl-148b	scr-148b	pre-ctrl	pre-148b
miR-148b-3p	32.901 ± 0.038	32.941 ± 0.163	33.389 ± 0.218	33.196 ± 0.562	28.653 ± 0.324	18.052 ± 0.21
U44	27.386 ± 0.092	27.55 ± 0.501	27.648 ± 0.084	27.929 ± 0.267	27.167 ± 0.023	27.28 ± 0.294
ΔCt	5.515 ± 0.099	5.391 ± 0.527	5.741 ± 0.234	5.267 ± 0.622	1.486 ± 0.325	-9.228 ± 0.361

Fig. S1

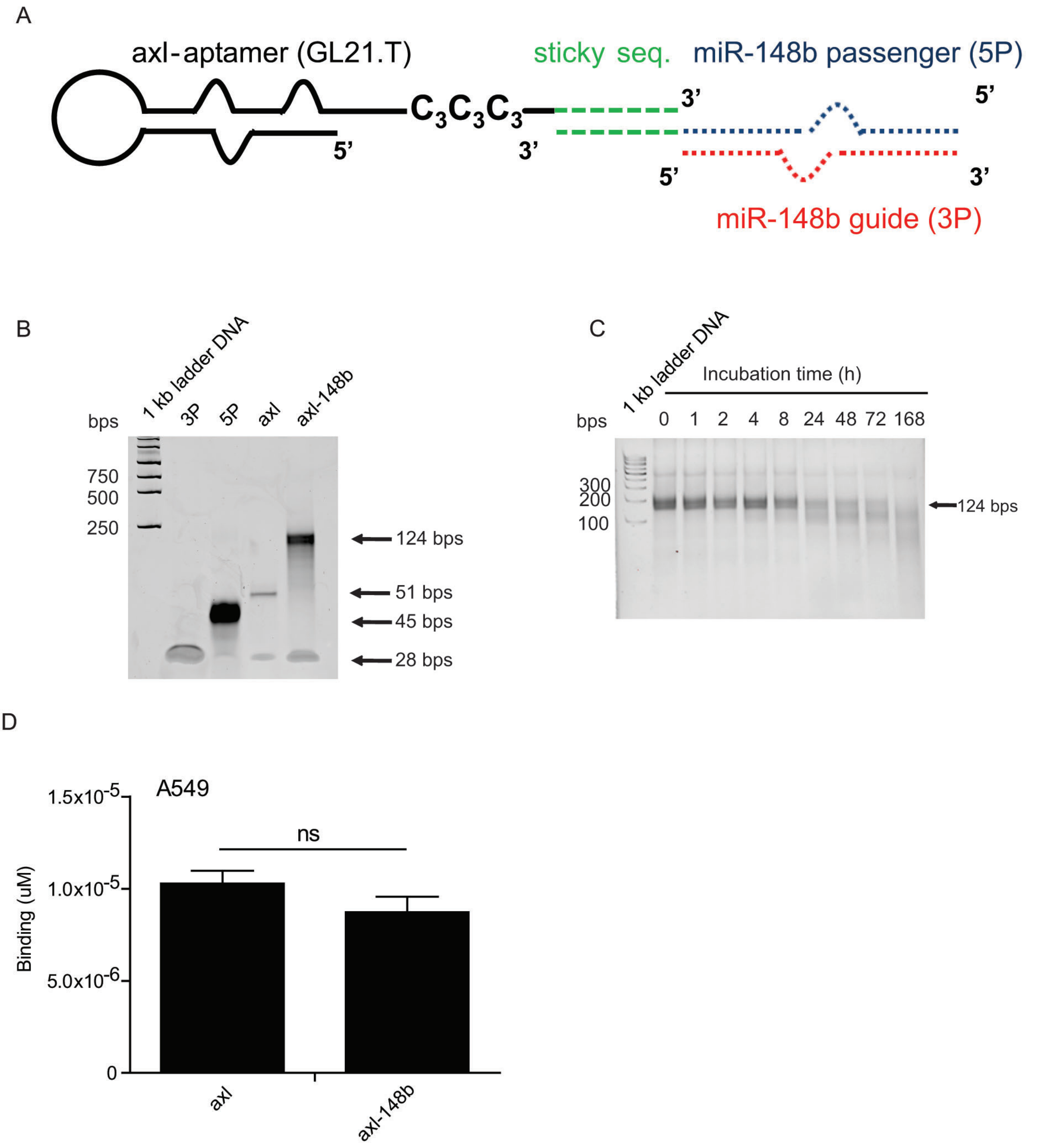
A
axl-aptamer (GL21.T)
sticky seq.
miR-148b passenger (5P)
C3C3C3
3'
5'
5'
3'
5'
3'
miR-148b guide (3P)
B
1 kb ladder DNA
3P
5P
axl
axl-148b
bps
750
500
250
124 bps
51 bps
45 bps
28 bps
C
1 kb ladder DNA
Incubation time (h)
bps
0 1 2 4 8 24 48 72 168
300
200
100
124 bps
D
A549
1.5x10-5
1.0x10-5
5.0x10-6
0
Binding (uM)
ns
axl
axl-148b

Fig. S2

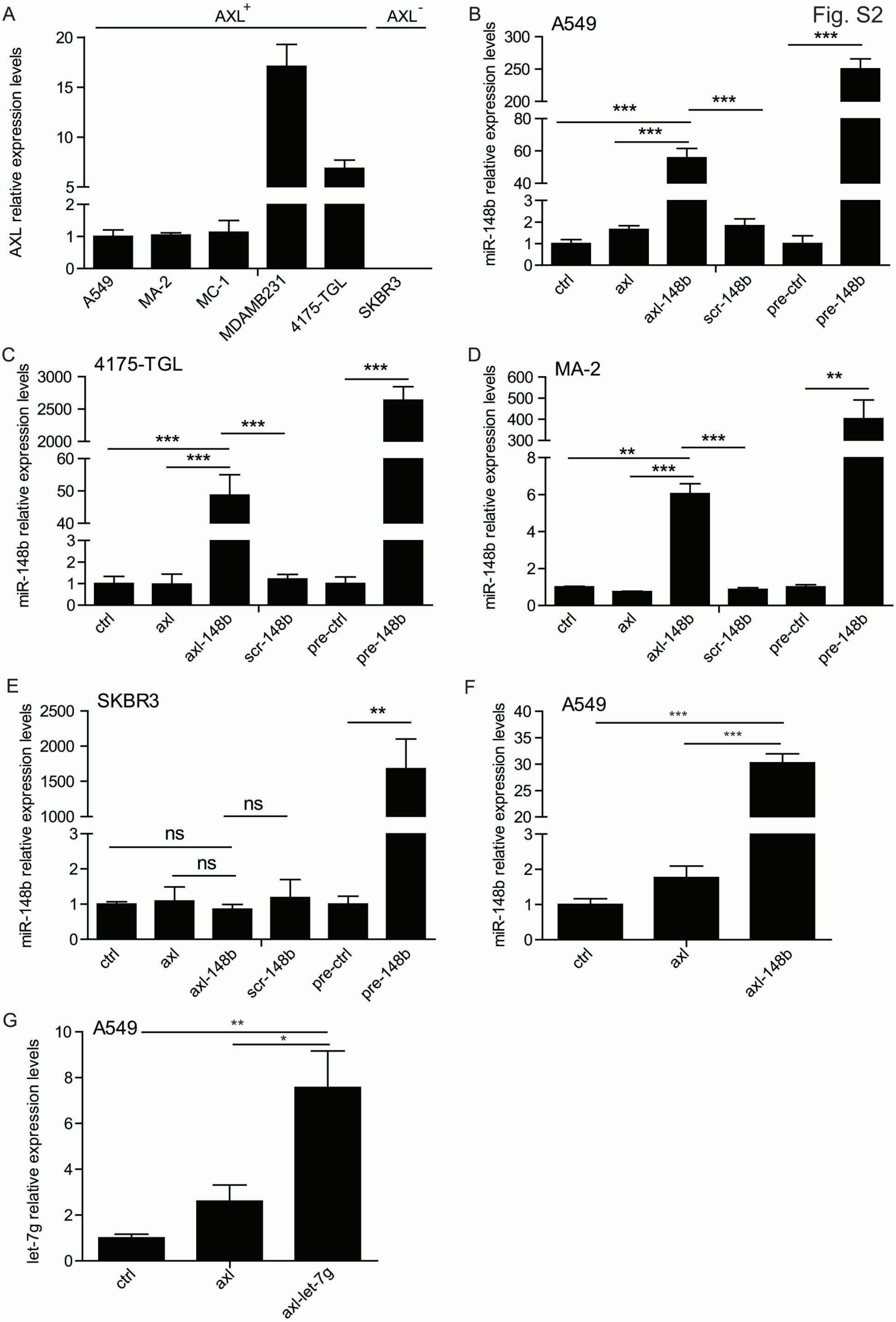

A
AXL+
AXL-
AXL relative expression levels
20
15
10
5
2
1
0
A549
MA-2
MC-1
MDAMB231
4175-TGL
SKBR3
B
A549
miR-148b relative expression levels
300
250
200
80
60
40
3
2
1
0
***
ctrl
axl
axl-148b
scr-148b
pre-ctrl
pre-148b
C
4175-TGL
miR-148b relative expression levels
3000
2500
2000
60
50
40
3
2
1
0
***
ctrl
axl
axl-148b
scr-148b
pre-ctrl
pre-148b
D
MA-2
miR-148b relative expression levels
600
500
400
300
8
6
4
2
0
**
***
ctrl
axl
axl-148b
scr-148b
pre-ctrl
pre-148b
E
SKBR3
miR-148b relative expression levels
2500
2000
1500
1000
3
2
1
0
**
ns
ctrl
axl
axl-148b
scr-148b
pre-ctrl
pre-148b
F
A549
miR-148b relative expression levels
40
35
30
25
20
3
2
1
0
***
ctrl
axl
axl-148b
G
A549
let-7g relative expression levels
10
8
6
4
2
0
**
*
ctrl
axl
axl-let-7g

Fig. S3

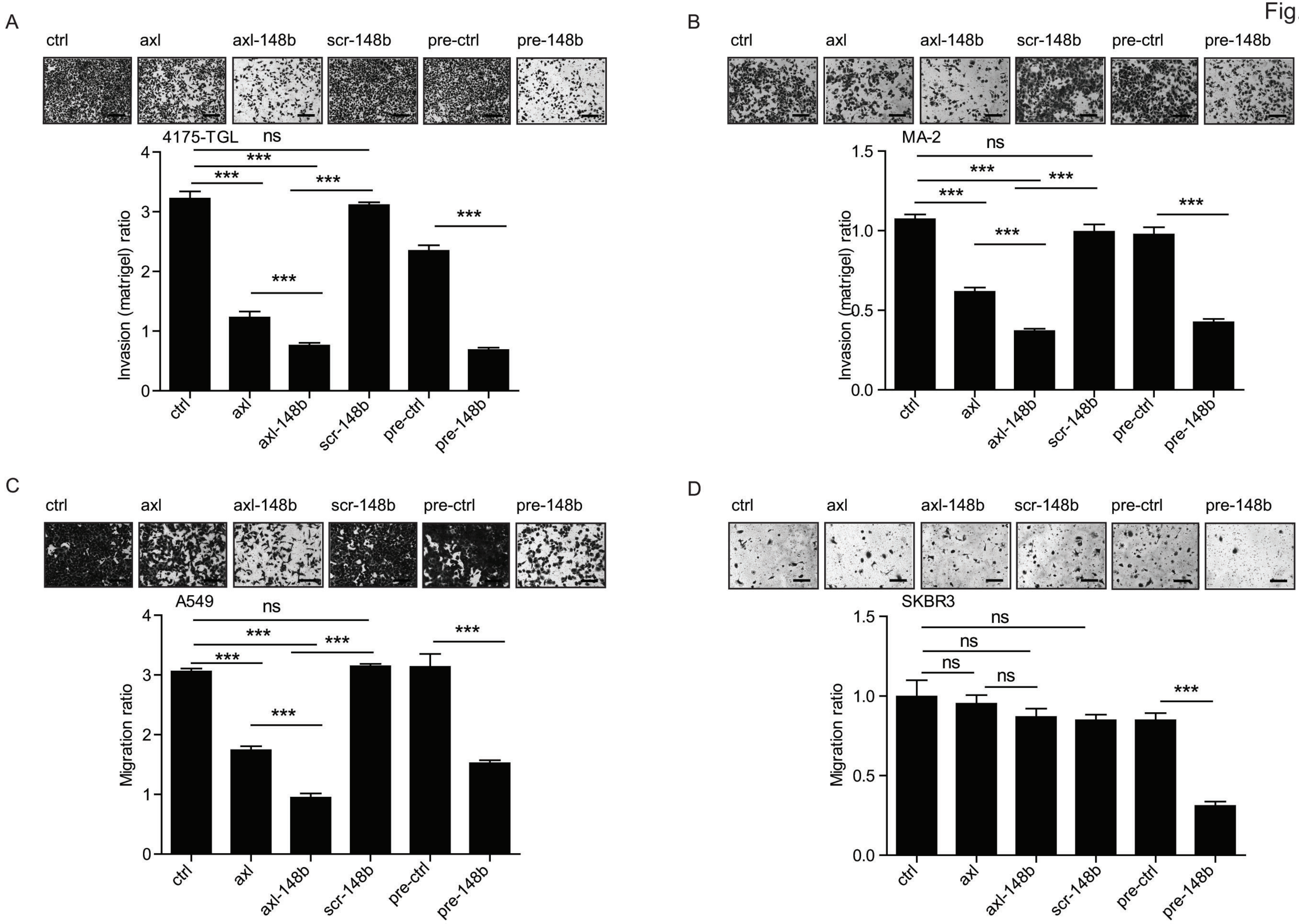

A
ctrl
axl
axl-148b
scr-148b
pre-ctrl
pre-148b
4175-TGL
ns
***
Invasion (matrigel) ratio
0
1
2
3
4
B
MA-2
Invasion (matrigel) ratio
0.0
0.5
1.0
1.5
C
A549
Migration ratio
D
SKBR3
Migration ratio

Fig. S4

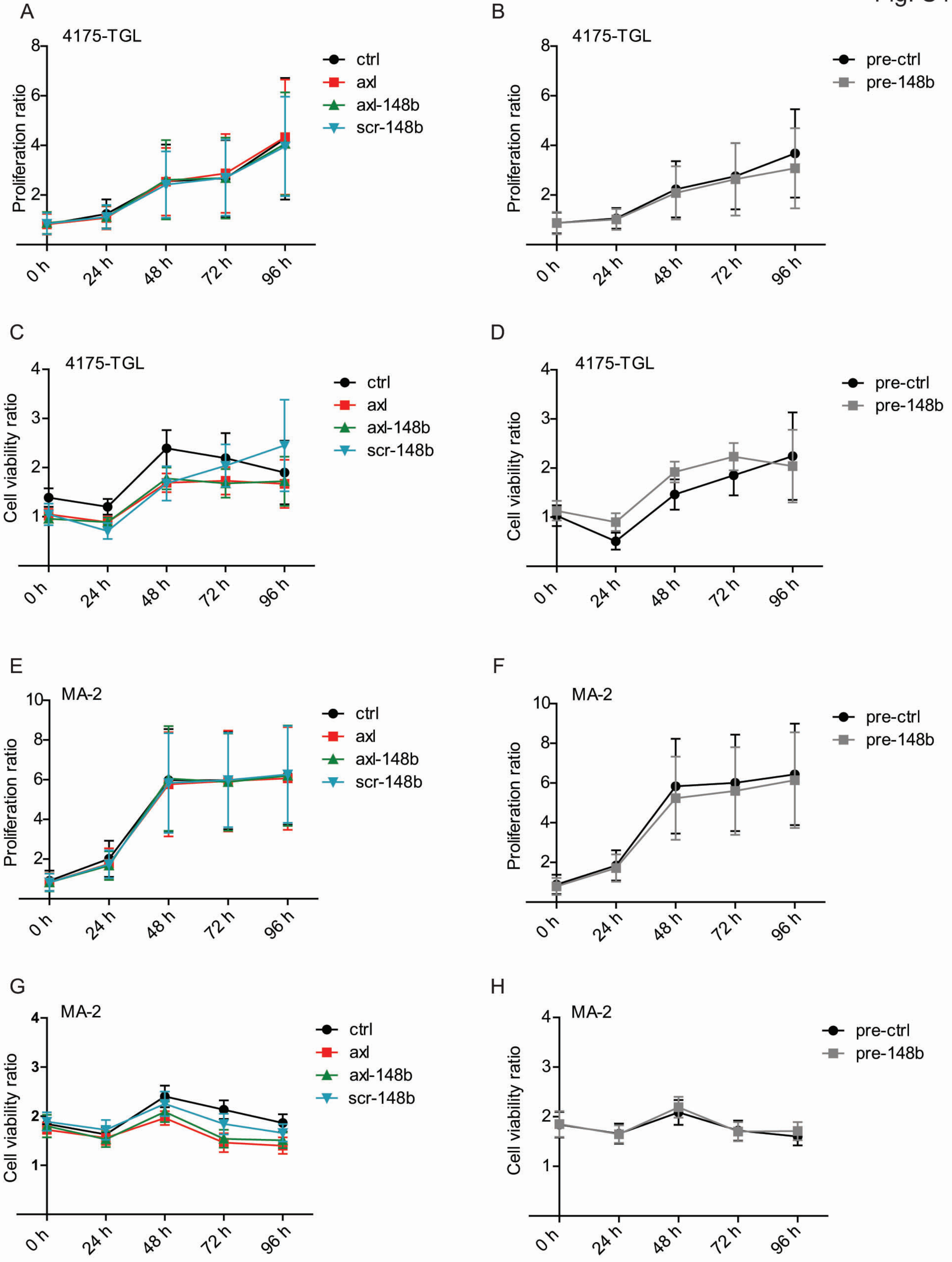
A
4175-TGL
Proliferation ratio
ctrl
axl
axl-148b
scr-148b
0 h
24 h
48 h
72 h
96 h
B
4175-TGL
Proliferation ratio
pre-ctrl
pre-148b
C
4175-TGL
Cell viability ratio
ctrl
axl
axl-148b
scr-148b
D
4175-TGL
Cell viability ratio
pre-ctrl
pre-148b
E
MA-2
Proliferation ratio
ctrl
axl
axl-148b
scr-148b
F
MA-2
Proliferation ratio
pre-ctrl
pre-148b
G
MA-2
Cell viability ratio
ctrl
axl
axl-148b
scr-148b
H
MA-2
Cell viability ratio
pre-ctrl
pre-148b

Fig. S5

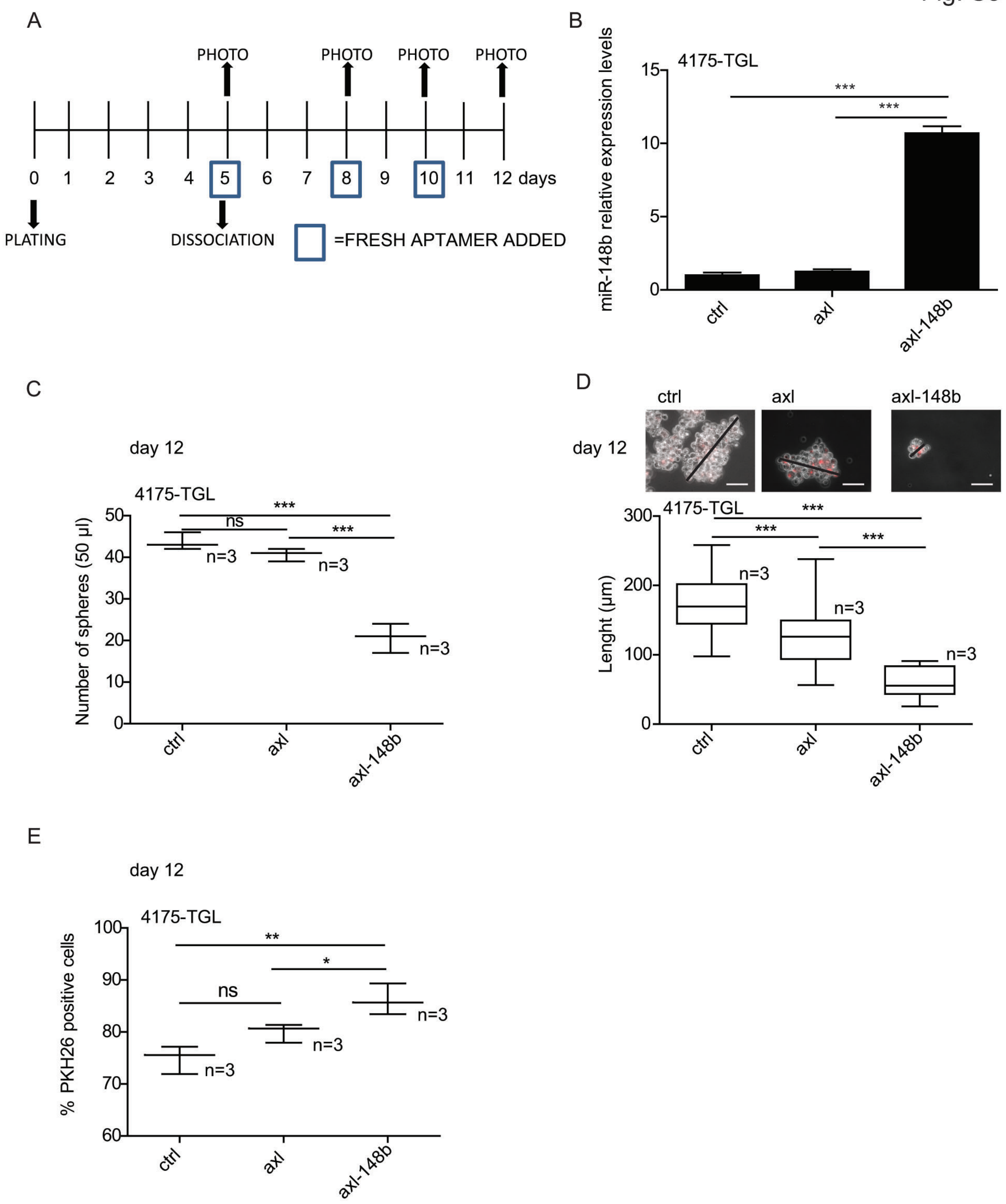
A
PHOTO
PHOTO
PHOTO
PHOTO
0 1 2 3 4 5 6 7 8 9 10 11 12 days
PLATING
DISSOCIATION
=FRESH APTAMER ADDED
B
4175-TGL
miR-148b relative expression levels
***
***
ctrl
axl
axl-148b
C
day 12
4175-TGL
Number of spheres (50 µl)
ns
***
***
n=3
ctrl
axl
axl-148b
D
ctrl
axl
axl-148b
day 12
4175-TGL
Lenght (µm)
***
***
***
n=3
E
day 12
4175-TGL
% PKH26 positive cells
**
*
ns
n=3

Fig. S6

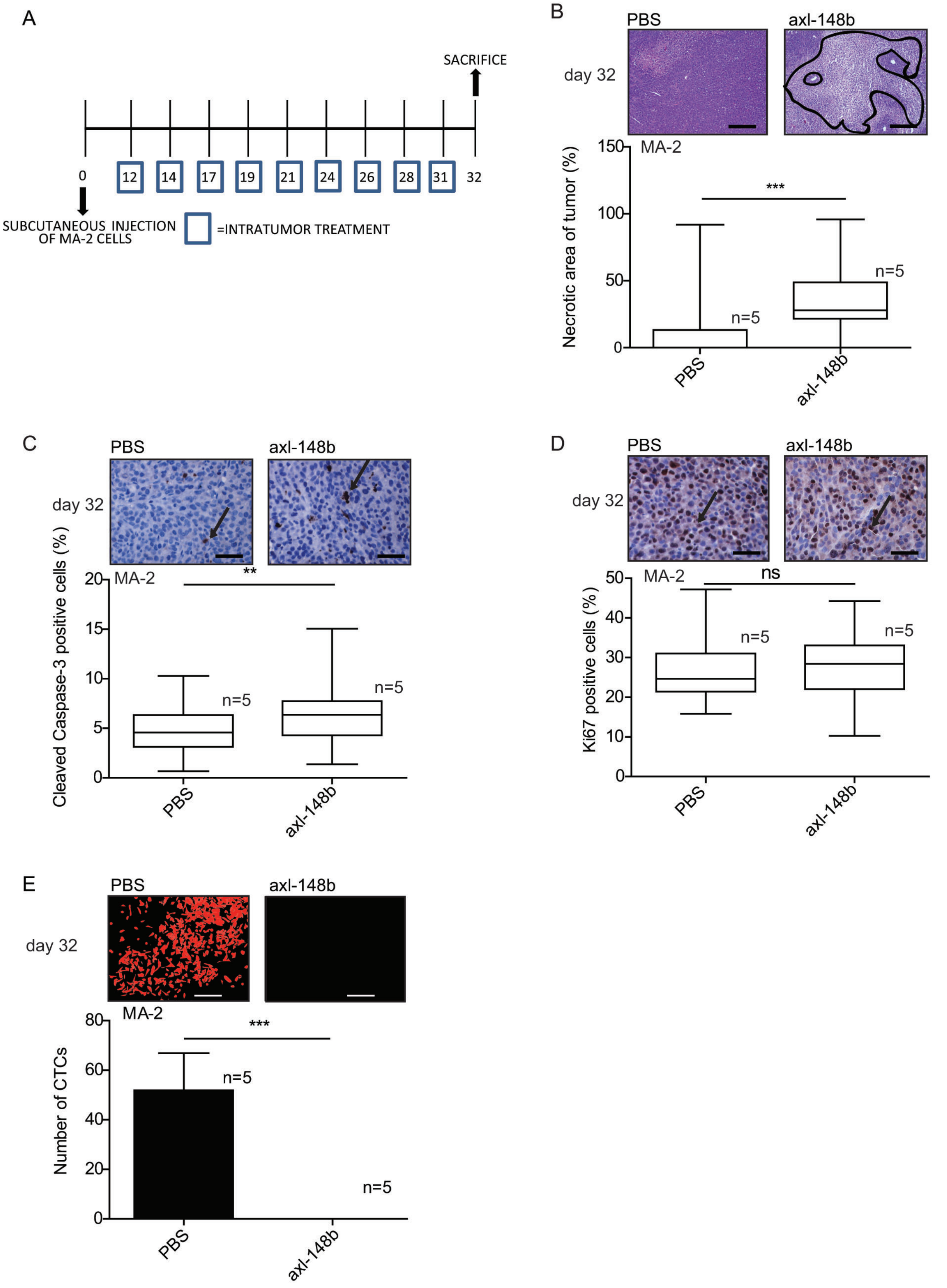

A
SACRIFICE
0
12
14
17
19
21
24
26
28
31
32
SUBCUTANEOUS INJECTION
OF MA-2 CELLS
=INTRATUMOR TREATMENT
B
PBS
axl-148b
day 32
MA-2
Necrotic area of tumor (%)
150
100
50
0
***
n=5
n=5
PBS
axl-148b
C
PBS
axl-148b
day 32
MA-2
Cleaved Caspase-3 positive cells (%)
20
15
10
5
0
**
n=5
n=5
PBS
axl-148b
D
PBS
axl-148b
day 32
MA-2
ns
Ki67 positive cells (%)
50
40
30
20
10
0
n=5
n=5
PBS
axl-148b
E
PBS
axl-148b
day 32
MA-2
Number of CTCs
80
60
40
20
0
***
n=5
n=5
PBS
axl-148b

Fig. S7

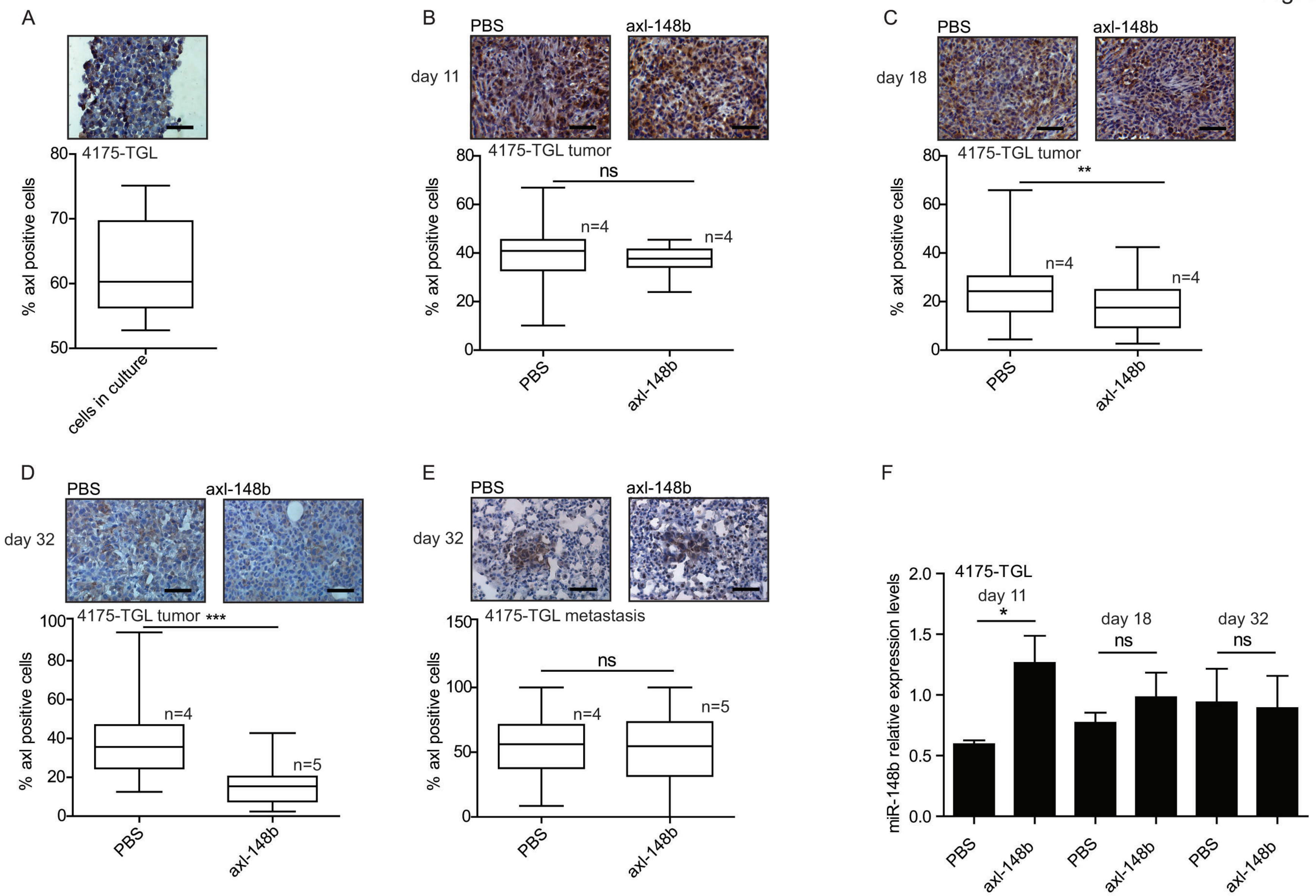
A
4175-TGL
% axl positive cells
cells in culture
B
PBS
axl-148b
day 11
4175-TGL tumor
ns
n=4
n=4
% axl positive cells
PBS
axl-148b
C
PBS
axl-148b
day 18
4175-TGL tumor
**
n=4
n=4
% axl positive cells
PBS
axl-148b
D
PBS
axl-148b
day 32
4175-TGL tumor ***
n=4
n=5
% axl positive cells
PBS
axl-148b
E
PBS
axl-148b
day 32
4175-TGL metastasis
ns
n=4
n=5
% axl positive cells
PBS
axl-148b
F
4175-TGL
day 11
*
day 18
ns
day 32
ns
miR-148b relative expression levels
PBS
axl-148b
PBS
axl-148b
PBS
axl-148b

Fig. S8

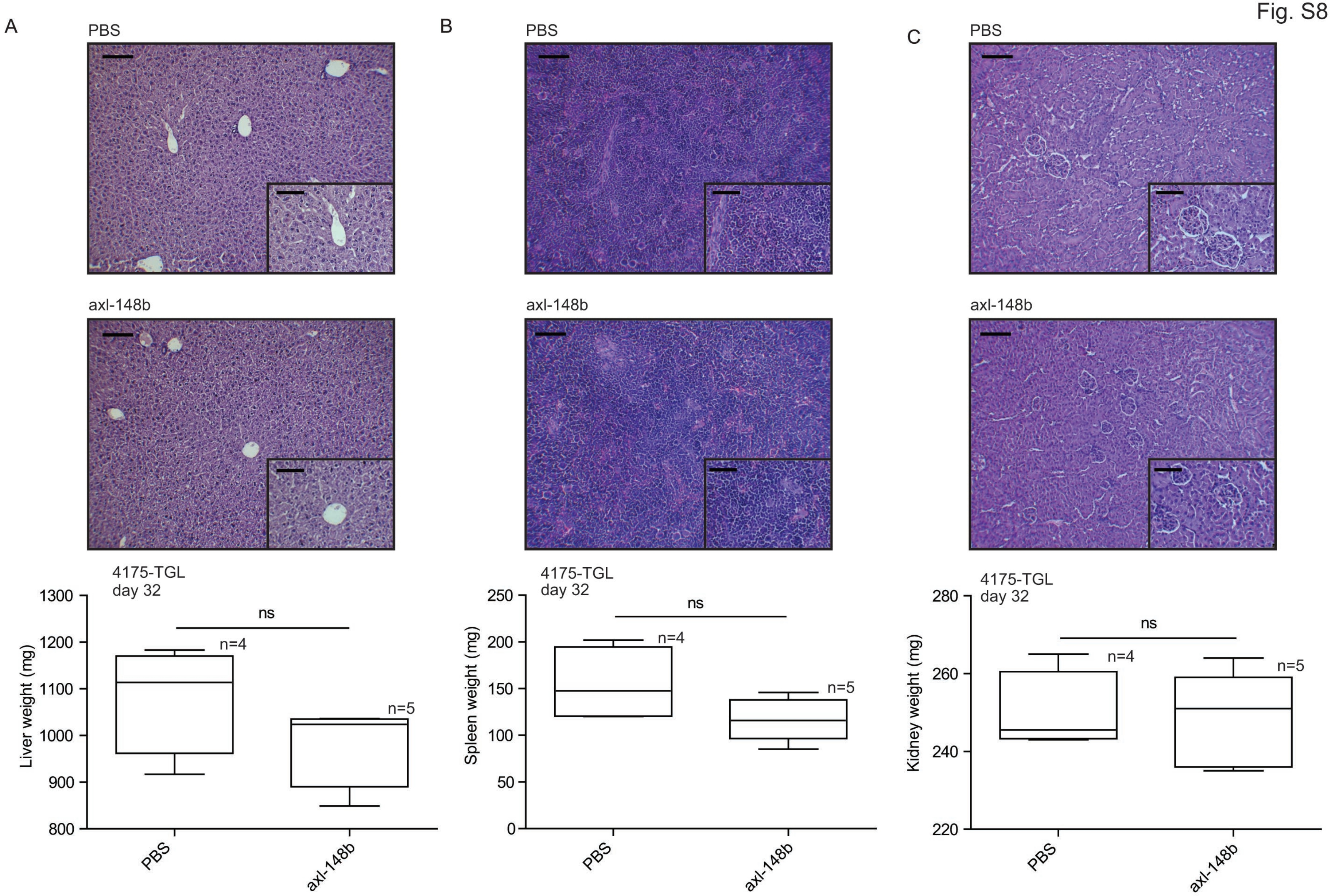
A
PBS
axl-148b
4175-TGL
day 32
ns
n=4
n=5
Liver weight (mg)
1300
1200
1100
1000
900
800
PBS
axl-148b
B
PBS
axl-148b
4175-TGL
day 32
ns
n=4
n=5
Spleen weight (mg)
250
200
150
100
50
0
PBS
axl-148b
C
PBS
axl-148b
4175-TGL
day 32
ns
n=4
n=5
Kidney weight (mg)
280
260
240
220
PBS
axl-148b
